# Supplementary material for: Warfarin analogs target disulfide bond-forming enzymes and suggest a residue important for quinone and coumarin binding
Source: J Biol Chem. 2024 May 16;300(6):107383. doi: 10.1016/j.jbc.2024.107383 (PMC11208910; doi:10.1016/j.jbc.2024.107383)
Supplement: Supplemental Figures S1–S3 [file mmc1.pdf]

Supporting Information

Title: **Warfarin analogs target disulfide bond-forming enzymes and suggest a residue important for quinone and coumarin binding**

Running Title: Targeting *de novo* disulfide bond formation

Dariana Chavez[1#], Gwendolyn Nita Amarquaye[1#], Adrian Mejia-Santana[1], Dyotima[1], Kayley Ryan[1], Lifan Zeng[2], Cristina Landeta[1*]

Author's affiliations:

[1] Department of Biology. Indiana University. Bloomington, USA.

[2] Indiana University Chemical Genomics Core Facility. Department of Biochemistry and Molecular Biology, School of Medicine, Indiana University. Indianapolis, USA.

[#] Authors contributed equally to this work

*Correspondence should be addressed to Dr. Cristina Landeta: clandeta@iu.edu

Supplementary Figure 1. Spacing is also important in addition to removal of two positive charges for *Hs*VKOR to complement the *E. coli* Δ*dsbB* mutant. Swarming motility was done by stabbing soft minimal M63 glucose plates (0.3% agar) supplemented with 10 μM IPTG. Motility halos of variants were measured after 48 h incubation at 30ºC. Data represents average±SD of at least three independent experiments.

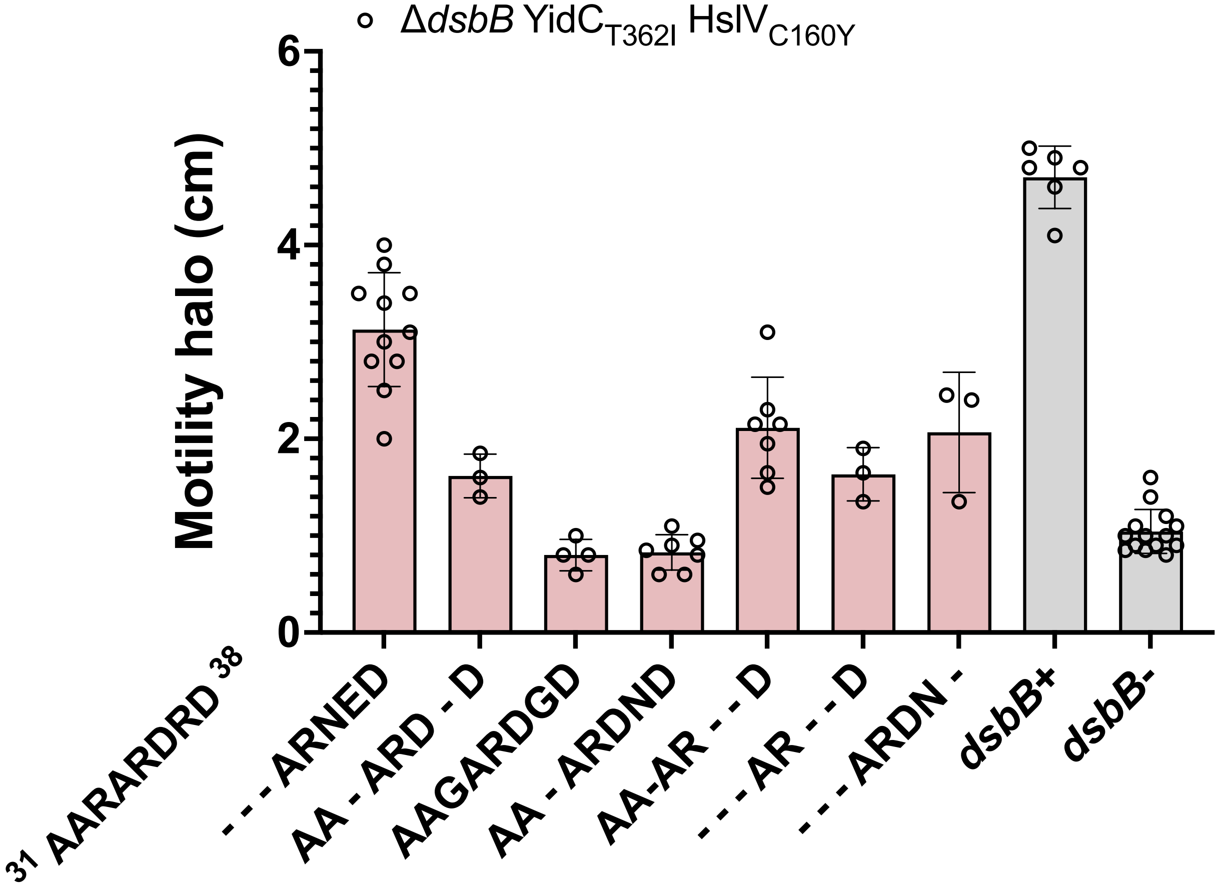

Supplementary Figure 2. Overexpression of *Ec*DsbB$_{R48Y}$, *Mt*VKOR$_{W146Y/F}$, *Hs*VKOR$_{Y139W}$ allows complementation of the *E. coli* Δ*dsbB* mutant. A) *E. coli* β-Gal^{dbs} Δ*dsbB* strains expressing DsbB or VKOR variants were grown on X-Gal M63 0.2% glucose media supplemented with various concentrations of IPTG at 30ºC for 24 h growth. A representative image is shown. The red dotted square indicates strains that grow poorly at those concentrations of IPTG*.* B) Swarming motility was done by stabbing soft minimal M63 glucose plates (0.3% agar) supplemented with various concentrations of IPTG. Halos were measured after 48 h incubation at 30ºC. Data represents average±SD of at least two independent experiments. C) Anaerobic growth of *E. coli* strains expressing DsbB or VKOR variants. Strains were grown aerobically to mid-log phase in M63 0.2% glucose. Cells were then diluted to an OD600 of 0.0001 (~1x10^5 CFU/mL) into 200 µL of anaerobic M63 0.2% glucose medium containing 100 mM potassium nitrate, and various concentrations of IPTG. Bacteria were enumerated after 24 h of anaerobic incubation at 37ºC.

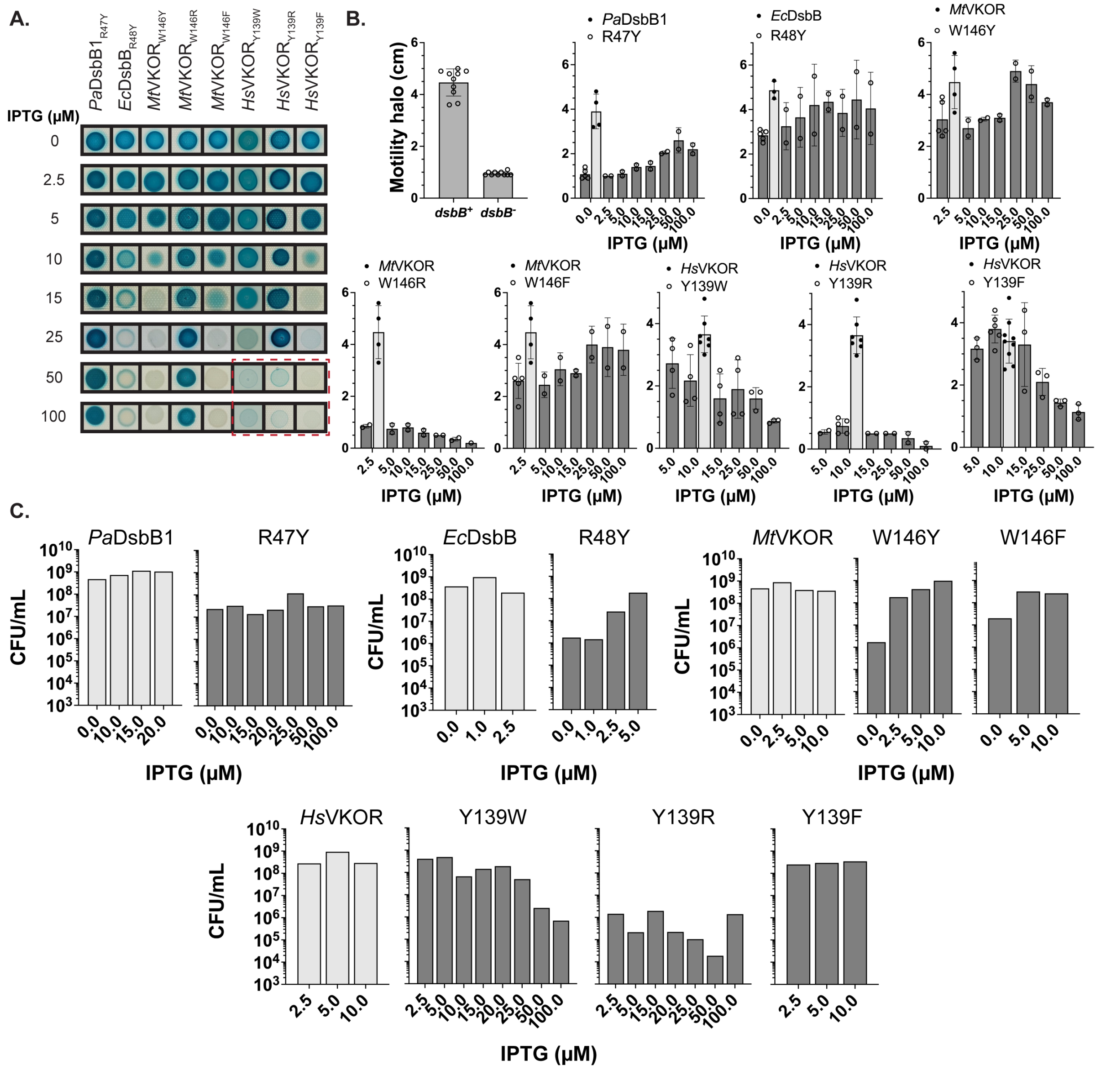

Supplementary Figure 3. CXXC+4 mutants are sensitized to 4-chloro-coumarin analogs with substitutions at position 3. Strains were grown aerobically to mid-log phase in M63 0.2% glucose. Cells were then diluted to an OD600 of 0.0001 (~1x10^5 CFU/mL, gray dotted line) into 200 µL of anaerobic M63 0.2% glucose medium containing 100 mM potassium nitrate and various concentrations of IPTG: 2.5 µM for *Mt*VKOR$_{W146Y}$, *Hs*VKOR and *Hs*VKOR$_{Y139W/F}$; 5 µM for *Ec*DsbB$_{R48Y}$ and *Mt*VKOR$_{W146R/F}$; 15 µM for *Hs*VKOR$_{Y139R}$; 25 µM for *Pa*DsbB$_{R47Y}$ and no addition of IPTG for *Ec*DsbB, *Pa*DsbB, and *Mt*VKOR. DMSO-diluted drugs were added to one final concentration indicated on the left with a 0.25% final DMSO concentration. Bacteria were enumerated after 24 h of anaerobic incubation at 37ºC. The results represent the average of at least three independent experiments.

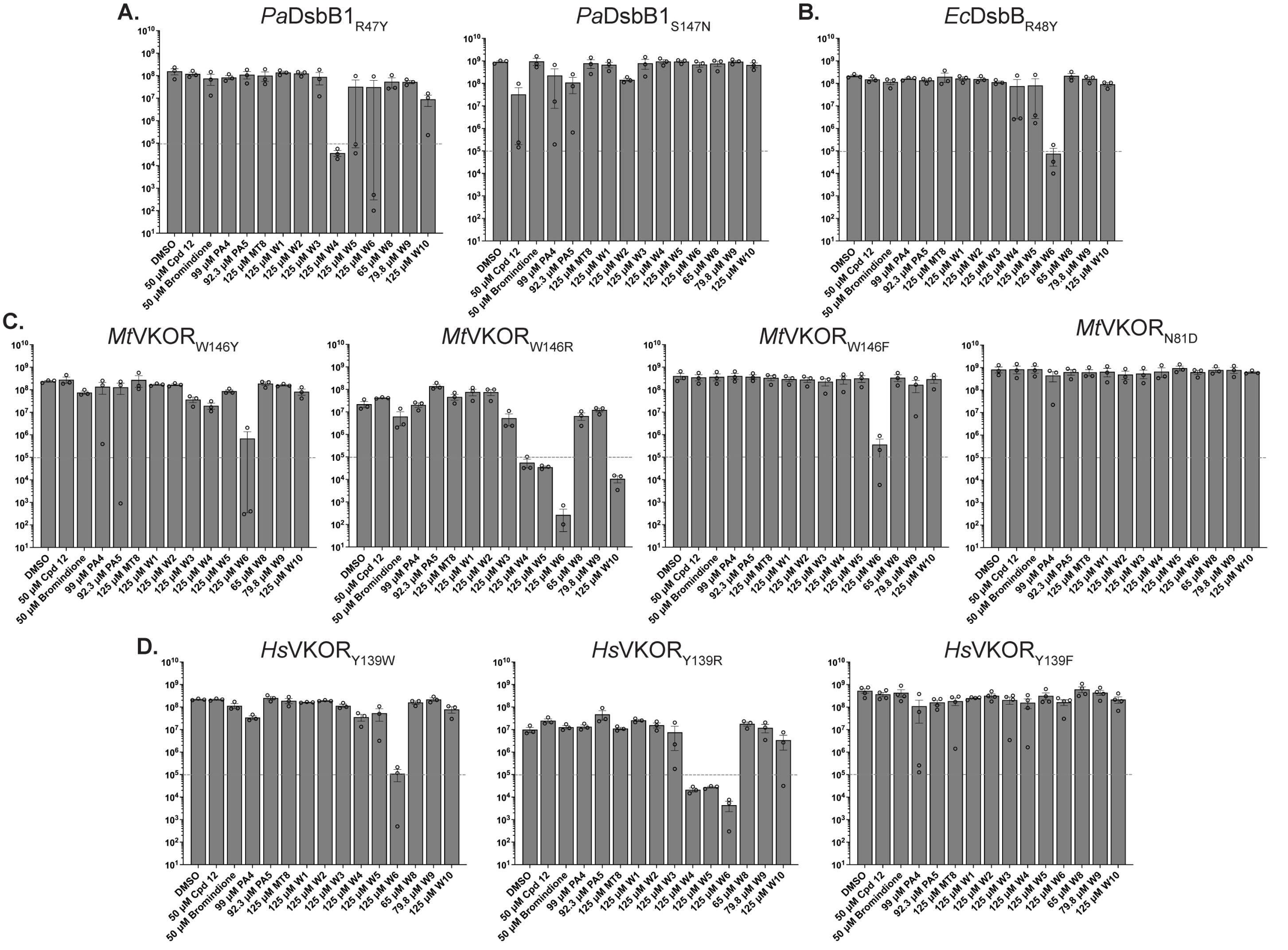

Full western blot images unedited:

Figure 1d

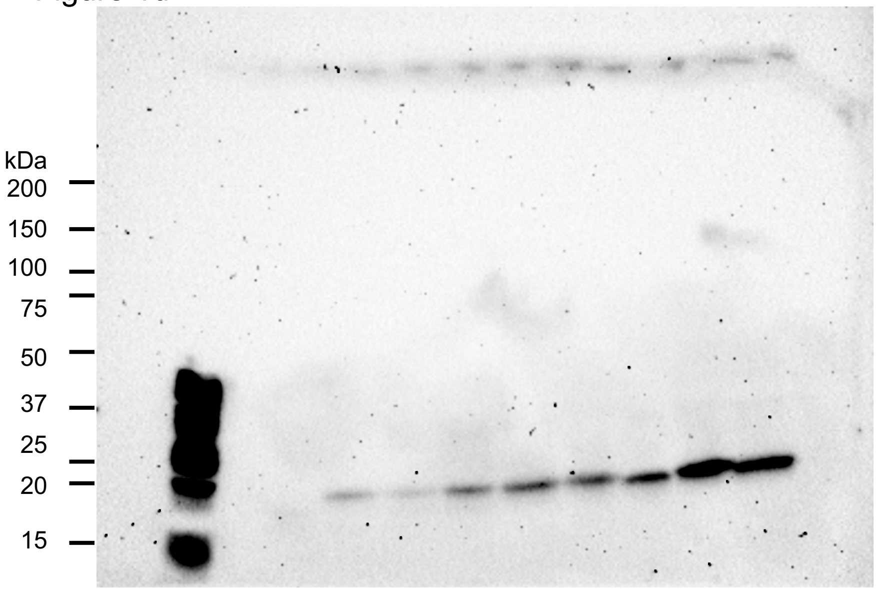

Figure 3b:

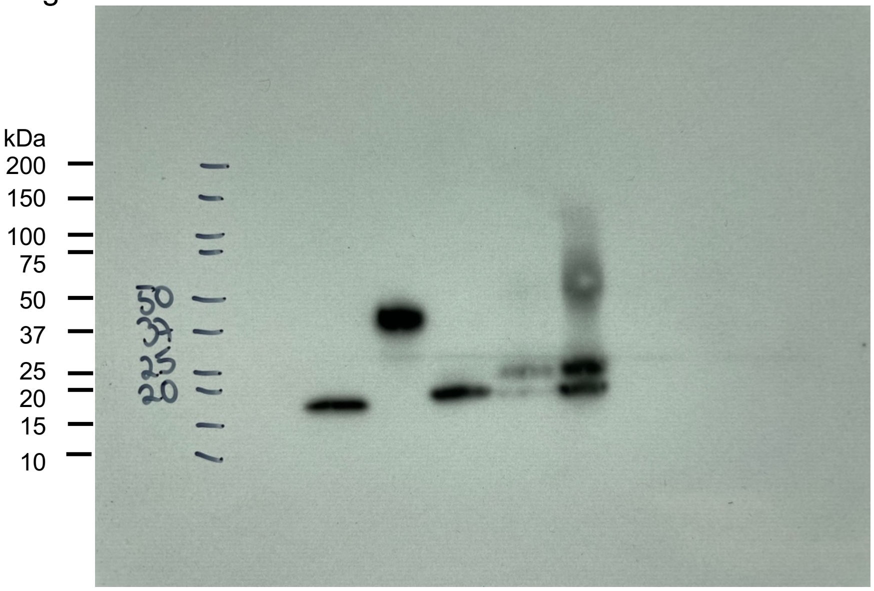

Figure 4f left:

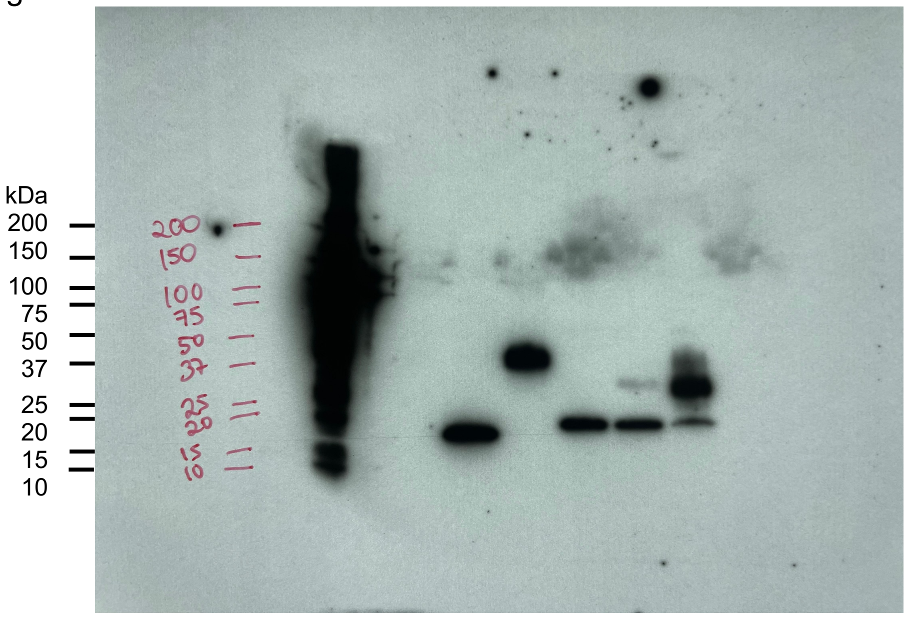

Figure 4f center:

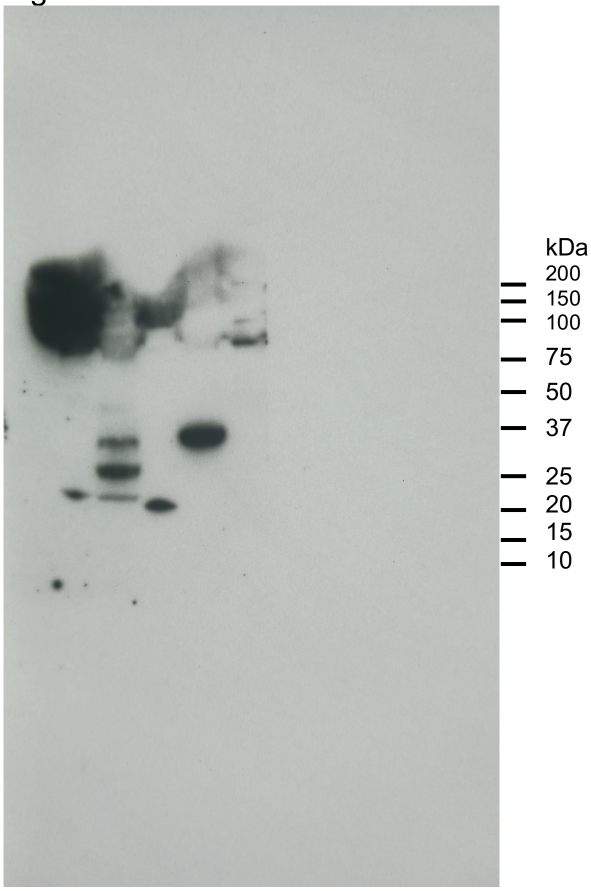

Figure 4f right:

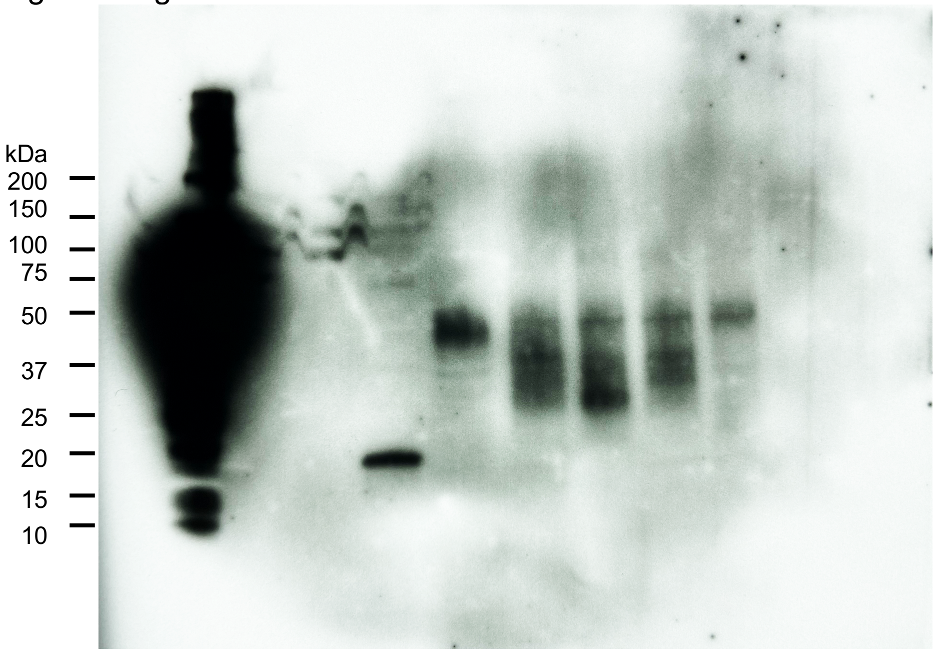

Additional supplementary information include:

1. HNMR and HPLC spectra of compounds W1 to W10

SYNTHESIS OF COMPOUNDS W1-W10

Synthesis of Compounds W1 (1), W2 (2) and W3 (3)

Bioduro-Sundia MediTech Company Ltd.

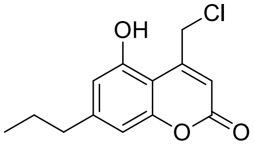

1

25 mg

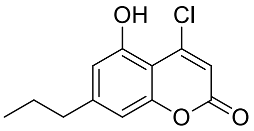

2

15 mg

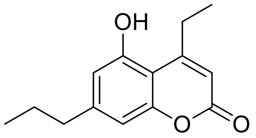

3

25 mg

Procedures:

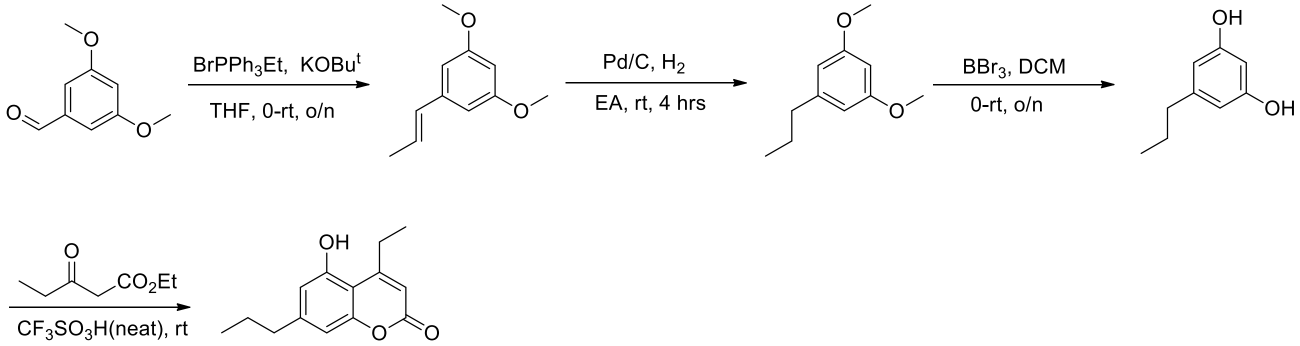

Step 1

To a solution of 3,5-dimethoxybenzaldehyde (1.66 g, 10.0 mmol) in THF (30 mL) was added Potassium tert-butanolate (1.68 g, 15.0 mmol) at 0°C. The resulting mixture was stirred from 0°C for 30 mins. Then ethyltriphenylphosphonium bromide was added and the mixture was stirred from 0°C to room temperature overnight. The reaction was monitored by LC-MS and TLC. Then the mixture was filtered, and the filtrate was concentrated *in vacuum* to give a residue, which was purified by silica gel column (PE/EtOAc = 100/1 to 30/1 as eluent) to afford 1,3-dimethoxy-5-(prop-1-en-1-yl)benzene (1.45 g, yield: 81 %) as colorless oil.

Step 2

To a solution of 1,3-dimethoxy-5-(prop-1-en-1-yl)benzene (1.45 g, 8.10 mmol) in EtOAc (40 mL) was added Pd/C (145 mg, 10% wt). The resulting mixture was stirred at room temperature for 4 hrs. The reaction was monitored by LC-MS. Then the mixture was filtered and the filtrate was concentrated *in vacuum* to give 1,3-dimethoxy-5-propylbenzene (1.46 g, yield: 99%) as colorless oil.

Step 3

To a solution of 1,3-dimethoxy-5-propylbenzene (1.46 g, 8.10 mmol) in dry DCM (30 mL) was added BB_{r3} (3.0 mL) at $0^{o}C$ dropwise. The resulting mixture was stirred from $0^{o}C$ to room temperature overnight. The reaction was monitored by LC-MS. Then the mixture was quenched by addition of MeOH (5.0 mL) slowly. The mixture was concentrated *in vacuum* to give a residue, which was purified by reverse-phase column (5-95% ACN in H_2O, 40 mins) to afford 5-propylbenzene-1,3-diol (1.18 g, yield: 96%) as yellow oil.

Step 4

To a mixture of 5-propylbenzene-1,3-diol (152 mg, 1.0 mmol) and ethyl 3-oxopentanoate (2.0 mL) was added CF_3SO_2OH (2 drops). The resulting mixture was stirred at room temperature overnight. The reaction was monitored by LC-MS. Then MeOH (5 mL) was added and the mixture was concentrated in vacuum to give a residue, which was purified by prep-HPLC (NH_4AC as additive) to afford 4-ethyl-5-hydroxy-7-propyl-2H-chromen-2-one (133.8 mg, yield: 58 %) as white solid.

^{1}HNMR (400 MHz, DMSO-*d6*): δ = 10.59 (brs, 1H), 6.66 (d, *J* = 1.2 Hz, 1H), 6.61 (d, *J* = 1.2 Hz, 1H), 6.04 (s, 1H), 2.98 (q, *J* = 7.6 Hz, 2H), 2.53 (t, *J* = 7.2 Hz, 2H), 1.65-1.53 (m, 2H), 1.19 (t, *J* = 7.6 Hz, 3H), 0.90 (t, *J* = 7.2 Hz, 3H). MS: m/z 233.1 ($M+H^+$).

Y1617-14835-017-01_HNMR(DMSO-d6, 40

Y1617-14835-017-01_LCMS.pdf

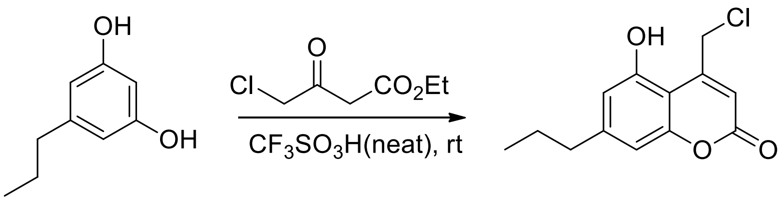

4-(chloromethyl)-5-hydroxy-7-propyl-2H-chromen-2-one

The title compound was prepared identical to the last step procedure of 4-ethyl-5-hydroxy-7-propyl-2H-chromen-2-one.

^{1}HNMR (400 MHz, DMSO-*d6*): δ = 10.91 (brs, 1H), 6.70 (s, 1H), 6.62 (d, *J* = 1.2 Hz, 1H), 6.42 (s, 1H), 5.09 (s, 2H), 2.54 (t, *J* = 7.2 Hz, 2H), 1.65-1.53 (m, 2H), 0.90 (t, *J* = 7.2 Hz, 3H). MS: m/z 253.1 ($M+H^+$).

Y1617-14835-018-01_HNMR(DMSO-d6, 40

Y1617-14835-018-01_LCMS.pdf

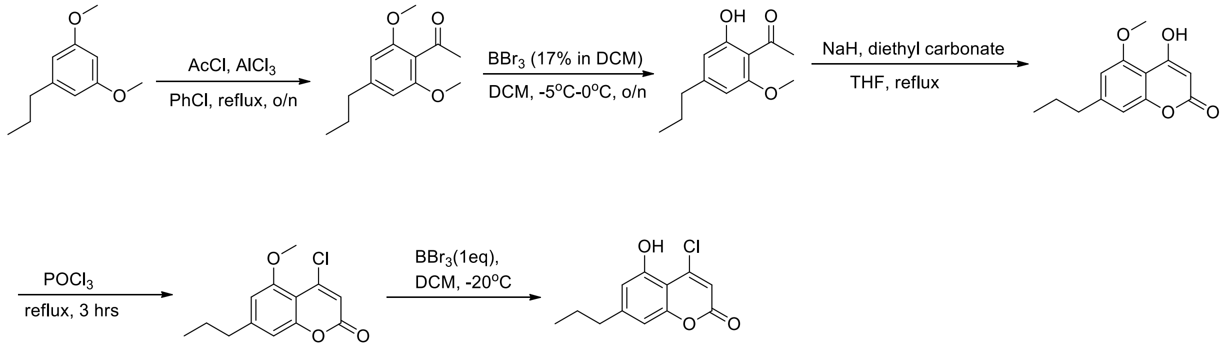

Step 1

To a solution of 1,3-dimethoxy-5-propylbenzene (460 mg, 2.56 mmol) in DCM (30 mL) was added acetyl chloride (300 mg, 3.83 mmol), followed by $AlCl_3$ (510 mg, 3.83 mmol) at 0°C. The resulting mixture was stirred at room temperature overnight. The reaction was monitored by LC-MS and TLC. Then the mixture was filtered and the filtrate was concentrated *in vacuum* to give a residue, which was purified by silica gel column (PE/EtOAc = 100/1 to 20/1) to afford 1-(2,6-dimethoxy-4-propylphenyl)ethanone (340 mg, yield: 60%) as yellow oil.

^{1}HNMR (400 MHz, DMSO-*d6*): δ = 6.37 (s, 2H), 3.79 (s, 6H), 2.56 (t, *J* = 8.0 Hz, 2H), 2.47 (s, 3H), 1.68-1.61 (m, 2H), 0.96 (t, *J* = 7.2 Hz, 3H).

E01617-14835-026-
02_HNMR(CDCl 3, 400

Step 2

To a solution of 1-(2,6-dimethoxy-4-propylphenyl)ethanone (340 mg, 1.53 mmol) in dry DCM (30 mL) was added BBr3 (2.0 mL, 17% in DCM) at 0°C dropwise. The resulting mixture was stirred from 0°C to room temperature overnight. The reaction was monitored by LC-MS. Then the reaction was quenched by addition of MeOH (5.0 mL) slowly. The mixture was concentrated *in vacuum* to give a residue, which was purified by silica gel column (PE/EtOAc = 100/1 to 20/1) to afford 1-(2-hydroxy-6-methoxy-4-propylphenyl)ethanone (242 mg, yield: 76%) as yellow oil.

Step 3

To a mixture of 1-(2-hydroxy-6-methoxy-4-propylphenyl)ethanone (192 mg, 0.92 mmol) in dry THF (20 mL) was added NaH (110 mg, 2.76 mmol). The mixture was stirred at 65°C for 10 mins and diethyl carbonate (218 mg, 1.84 mmol) was added drop wise. The resulting mixture was stirred at 65°C overnight. The reaction was monitored by LC-MS. Then MeOH (5 mL) was added to quench the reaction and the mixture was concentrated *in vacuum* to give a residue, which

was purified by silica gel column (DCM to DCM/MeOH = 30/1) to afford 4-hydroxy-5-methoxy-7-propyl-2H-chromen-2-one (148 mg, yield: 58 %) as white solid.

Step 4

A mixture of 4-hydroxy-5-methoxy-7-propyl-2H-chromen-2-one (148 mg, 0.63 mmol) in $POCl_3$ (10 mL) was stirred at 100°C for 3 hrs. The reaction was monitored by LC-MS. Then $POCl_3$ was removed *in vacuum* to give a residue, which was purified by silica gel column (DCM) to afford 4-chloro-5-methoxy-7-propyl-2H-chromen-2-one (62 mg, yield: 39 %) as white solid.

^{1}HNMR (400 MHz, DMSO-*d6*): δ = 6.79 (s, 1H), 6.60 (s, 1H), 6.39 (s, 1H), 3.92 (s, 3H), 2.64 (t, *J* = 7.6 Hz, 2H), 1.74-1.63 (m, 2H), 0.97 (t, *J* = 7.2 Hz, 3H).

E01617-14835-030-01_HNMR(CDCl3, 400

Step 5

To a solution of 4-chloro-5-methoxy-7-propyl-2H-chromen-2-one (62 mg, 0.25 mmol) in dry DCM (30 mL) was added BBr3 (1.0 mL, 17% in DCM) at 0°C drop wise. The resulting mixture was stirred from 0°C to room temperature overnight. The reaction was monitored by LC-MS. Then the reaction was quenched by addition of MeOH (2.0 mL) slowly. The mixture was concentrated *in vacuum* to give a residue, which was purified by Prep-TLC (DCM/MeOH = 30/1) to afford 4-chloro-5-hydroxy-7-propyl-2H-chromen-2-one (43.4 mg, yield: 74%) as white solid.

^{1}HNMR (400 MHz, DMSO-*d6*): δ = 10.84 (brs, 1H), 6.73 (d, *J* = 1.2 Hz, 1H), 6.65 (d, *J* = 1.2 Hz, 1H), 6.48 (s, 1H), 2.54 (d, *J* = 7.2 Hz, 2H), 1.64-1.54 (m, 2H), 0.89 (t, *J* = 7.2 Hz, 3H). MS: m/z 239.0 $(M+H^{+})$.

E01617-14835-031-01_HNMR(DMSO-d6, 4

E01617-14835-031-01_LCMS.pdf

Synthesis of Compound W4

Bioduro-Sundia MediTech Company Ltd.

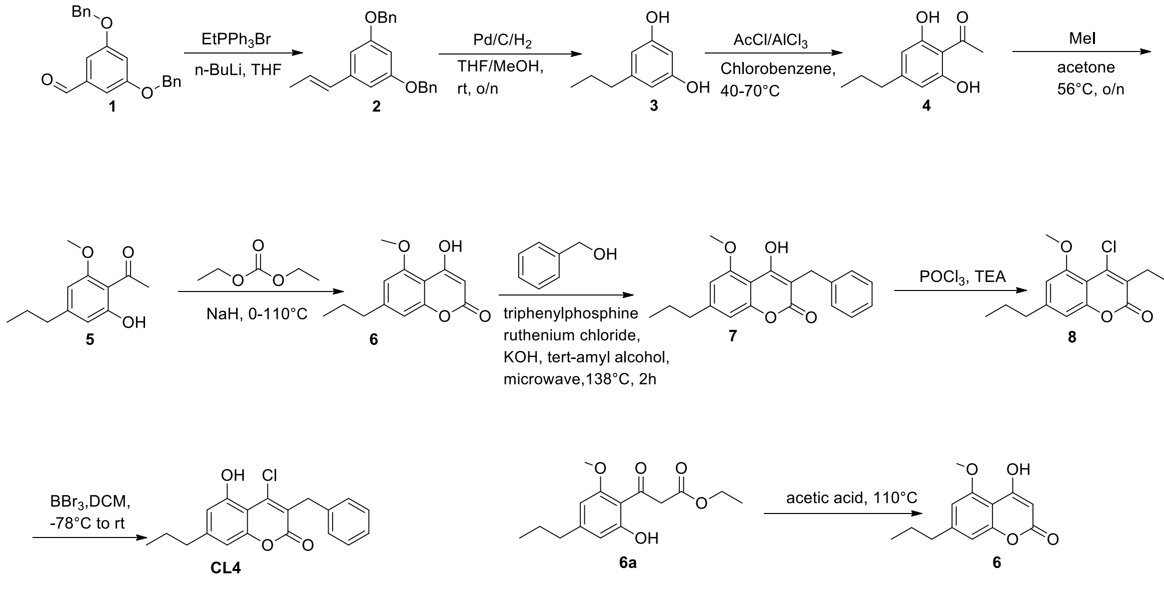

To a solution of ethyl triphenyl phosphanium bromide (32.06 g, 0.0863 mol) in THF (333 mL) was added n-butyllithium (5.53 g, 34 ml, 0.0863 mol) at 0 ℃, and the reaction mixture was stirred for half an hour at 0 ℃. Then 3,5-bis(benzyloxy)benzaldehyde **1** (25 g, 0.0785 mmol) was added. The reaction mixture was stirred at 25 ℃ for one hour. The reaction mixture was quenched with water (200 mL) and was extracted with EA (100 mL x 3). The combined organic layer was washed with water (100 mL) and brine (100 mL), dried over Na_2SO_4 and concentrated in vacuo. The residue was purified silica gel column chromatographed (PE/EA = 50/1) to afford compound **2** as yellow oil (13.01 g, yield: 48%). ^{1}H NMR (300 MHz, $CDCl_3$): *δ* 7.58-7.37 (m, 10H), 6.70-6.57 (m, 3H), 6.50-5.80 (m, 2H), 5.12 (s, 4H), 2.00-1.92 (m, 3H). LC-MS: m/z 331.1 $[M+H]^+$.

2_HNMR

To a solution of 1,3-bis(benzyloxy)-5-[(1E)-prop-1-en-1-yl]benzene **2** (13.009 g, 0.0394 mol) in THF (51 ml) and MeOH (173 ml) was added 10% Pd/C (2.6 g） at 25 ℃. The mixture was stirred under hydrogen balloon for 15 hours at room temperature. The reaction solution was filtered and the filtrate was concentrated to get crude compound **3** (5.8 g, yield: 92%) as a yellow solid. ^{1}H NMR (300 MHz, DMSO-d_6): *δ* 9.04 (s, 2H), 6.07 (s, 3H), 2.38 (t, *J* = 7.5 Hz, 2H), 1.58-1.50 (m, 2H), 0.89 (t, *J* = 7.2 Hz, 3H).

3_HNMR

To a solution of 5-propylbenzene-1,3-diol **3** (3.5 g, 0.023 mol), $AlCl_3$ (9.2 g 0.069 mol) in chlorobenzene (28 ml) was added AcCl（1.79 g 0.023 mol）at 40℃ and stirred for 30 minutes. Subsequently, the temperature was raised to 70 °C and the reaction mixture was stirred for an additional hour. The reaction was cooled to room temperature. The reaction mixture was quenched with water (50 mL) and 1M HCl (14 ml) was added until PH less than 1. The mixture was extracted with EA (50 mL x 3), the organics were washed with water (50 mL) and brine (50 mL), dried over Na_2SO_4. The organics were concentrated in vacuo and the residue was purified silica gel column chromatographed (PE/EA = 30/1) to afford compound **4** as a yellow solid (2.6 g, yield: 53%). LC-MS: m/z 195.0 $[M+H]^+$.

Compound 4_LCMS.pdf

A solution of 1-(2,6-dihydroxy-4-propylphenyl) ethenone **4** (1.79 g, 0.0092 mol), K_2CO_3 (1.27 g 0.0092 mol), CH_3I（1.31 g 0.0092 mol）in acetone (15.3 ml) was added into a glass tube and sealed. The reaction mixture was stirred for 22 hours at 56 ℃. The mixture was filtered. Then, the filtrate was concentrated in vacuo and the residue was purified silica gel column chromatographed (PE) to afford compound **5** as a yellow oil (1.173 g, yield: 59%). LC-MS: m/z 209.0 $[M+H]^+$.

Compound 5_LCMS.pdf

To a solution of 1-(2-hydroxy-6-methoxy-4-propylphenyl) ethanone **5** (1.335 g, 0.0064 mol) in diethyl carbonate (30 ml) was added NaH (1.54 g, 0.064 mol) at 0 ℃. Subsequently, the temperature was raised to 110 °C and the reaction mixture was stirred for 30 minutes. The reaction was cooled to room temperature. The reaction mixture was quenched with water (30 mL) and 1M HCl (12 ml) was added. The mixture was extracted with EA (50 mL x 2), the organics were washed with water (50 mL) and brine (50 mL), dried over Na_2SO_4. The organics were concentrated in vacuo and the residue was purified silica gel column chromatograph (PE/EA = 4/1) to afford compound **6** as a white solid (470 mg, yield: 30%). LC-MS: m/z 235.0 $[M+H]^+$.

Compound 6_LCMS.pdf

A solution of 4-hydroxy-5-methoxy-7-propylchromen-2-one **6** (200 mg, 0.8538 mmol), Benzyl alcohol (92.26 mg 0.8538 mmol), triphenylphosphine ruthenium chloride（40.94 mg 0.0426 mmol), KOH (9.59 mg, 0.01707 mmol) in tert-amyl alcohol (2 ml) was added into a microwavable glass tube. Subject the mixture to microwave irradiation at 138 °C for 2 hours. The reaction mixture was quenched with water (10 mL) and extracted with EA (10 mL x 2), the

organics were washed with water (10 mL) and brine (10 mL), dried over Na_2SO_4. The organics were concentrated in vacuo and the residue was purified silica gel column chromatograph (PE/EA = 15/1) to afford compound **7** as a brown solid (118 mg, yield: 38%). LC-MS: m/z 325.0 $[M+H]^+$.

Compound 7_LCMS.pdf

To a solution of 3-benzyl-4-hydroxy-5-methoxy-7-propylchromen-2-one **7** (166 mg, 0.5118 mmol) in $POCl_3$ (3 ml) was added TEA (62.15 mg 0.6141 mmol). The reaction mixture was stirred for one hour at 70°C. The reaction mixture was quenched with $NaHCO_3$ (10 mL). The mixture was extracted with EA (10 mL x 3), the organics were washed with water (10 mL x 3) and brine (10 mL x 3), dried (Na_2SO_4). The organics were concentrated in vacuo and the residue was purified silica gel column chromatograph (PE/EA = 10/1) to afford compound **8** as a white solid (118 mg, yield: 64%). LC-MS: m/z 343.0 $[M+H]^+$.

Compound 6_LCMS.pdf

To a solution of 3-benzyl-4-chloro-5-methoxy-7-propylchromen-2-one **8** (118 mg, 0.344 mmol) in DCM (8.2 ml) was added BBr_3 (2M in DCM, 0.86 mL, 1.721 mmol) at -78°C. The reaction mixture was allowed to warm to room temperature and stirred for 4 hours. The reaction mixture was quenched with water (10 mL) and $NaHCO_3$ (6 mL). The mixture was extracted with EA (10 mL x 2), the organics were washed with water (10 mL) and brine (10 mL), dried over Na_2SO_4. The organics were concentrated in vacuo and the residue was purified by Prep-HPLC (0.1% NH_4HCO_3 in H_2O/MeCN) to afford CL4 as a white solid (50 mg, yield: 42%). ^1H NMR (400 MHz, CD_3OD): δ 7.32-7.17 (m, 5H), 6.81 (s, 1H), 6.68 (s, 1H), 3.94 (s, 2H), 2.65 (t, J = 7.2 Hz, 2H), 1.71-1.65 (m, 2H), 0.96 (t, J = 7.2 Hz, 3H). LC-MS: m/z 329.0 $[M+H]^+$.

CL4_HNMR CL4_LCMS

Synthesis of Compound W5

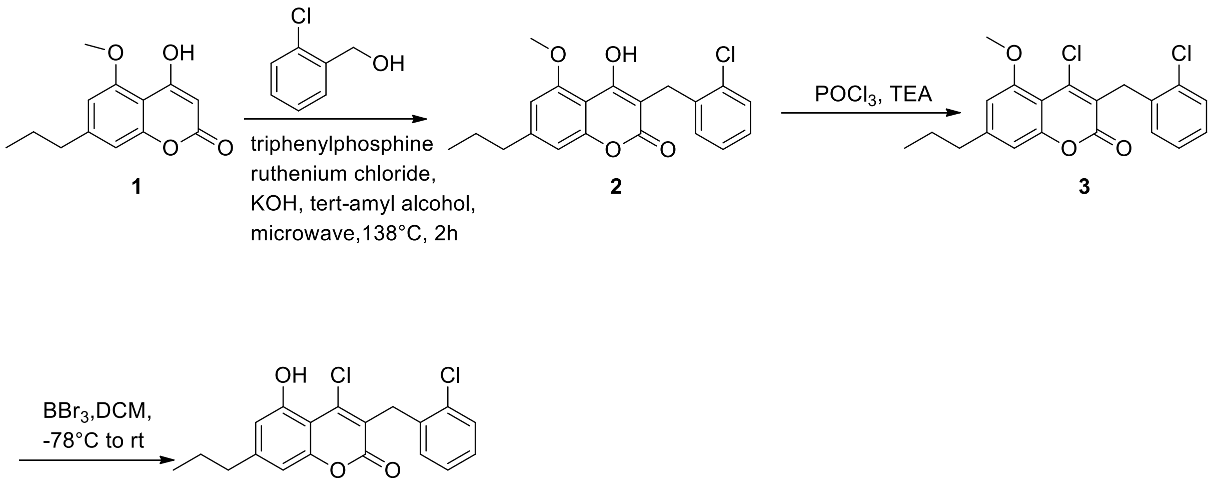

CL5

A solution of 4-hydroxy-5-methoxy-7-propylchromen-2-one **1** (620 mg, 0.265 mmol), 2-Chlorobenzyl alcohol (375 mg 0.265 mmol), triphenylphosphine ruthenium chloride（127 mg 0.0132 mmol), KOH (31 mg, 0.053 mmol) in tert-amyl alcohol (6.2 ml) was added into a microwavable glass tube. Subject the mixture to microwave irradiation at 138 °C for 2 hours. The reaction mixture was quenched with water (15 mL) and extracted with EA (10 mL x 2), the organics were washed with water (15 mL) and brine (15 mL), dried over Na_2SO_4. The organics were concentrated in vacuo and the residue was purified silica gel column chromatograph (PE/EA = 10/1) to afford compound **2** as a brown solid (329 mg, yield:35 %). ^{1}H NMR (400 MHz, DMSO-d_6): δ 10.18 (s, 1H), 7.29-7.10 (m, 4H), 6.93-6.89 (m, 2H), 4.01 (s, 3H), 3.84 (s, 2H), 2.66 (t, J = 7.2 Hz, 2H), 1.72-1.62 (m, 2H), 0.92 (t, J = 7.2 Hz, 3H). LC-MS: m/z 359.0 $[M+H]^+$.

2-HNMR.pdf

To a solution of 3-[(2-chlorophenyl)methyl]-4-hydroxy-5-methoxy-7-propylchromen-2-one **2** (308 mg, 0.8584 mmol) in $POCl_3$ (5 ml) was added TEA (104 mg 1.0301 mmol). The reaction mixture was stirred for one hour at 70°C. The reaction mixture was quenched with $NaHCO_3$ (10 mL). The mixture was extracted with EA (10 mL x 3), the organics were washed with water (10 mL) and brine (10 mL) and dried over Na_2SO_4. The organics were concentrated in vacuo and the residue was purified silica gel column chromatograph (PE/EA = 10/1) to afford compound **3** as a white solid (178 mg, yield:52%). LC-MS: m/z 376.9 $[M+H]^+$.

E03594-ENB2273
06-059-03.pdf

To a solution of 4-chloro-3-[(2-chlorophenyl)methyl]-5-methoxy-7-propylchromen-2-one **3** (178 mg, 0.4718 mmol) in DCM (13 ml) was added BBr_3 (2M in DCM, 1.2 mL, 2.359 mmol) at -78°C. The reaction mixture was allowed to warm to room temperature and stirred for 4 hours. The reaction mixture was quenched with water (10 mL) and $NaHCO_3$ (6 ml) was added. The mixture was extracted with EA (10 mL x 2), the organics were washed with water (10 mL x 3) and brine (10 mL x 3), dried (Na_2SO_4). The organics were concentrated in vacuo and purified by Prep-HPLC(NH_4HCO_3) to afford CL5 as a white solid (20 mg, yield:12%). ^{1}H NMR (400 MHz, $CDCl_3$): *δ* 12.06 (s, 1H), 7.40-7.37 (m, 1H), 7.18-7.12 (m, 2H), 7.07-7.04 (m, 1H), 6.74 (d, *J* = 1.6 Hz, 1H), 6.69 (d, *J* = 1.6 Hz, 1H), 4.07 (s, 2H), 2.64 (t, *J* = 7.2 Hz, 2H), 1.71-1.63 (m, 2H), 0.96 (t, *J* = 7.2 Hz, 3H). LC-MS: m/z 363.0 $[M+H]^+$.

CL5_HNMR　CL5-HPLC.pdf　CL5-LCMS.pdf

Synthesis of Compound W6

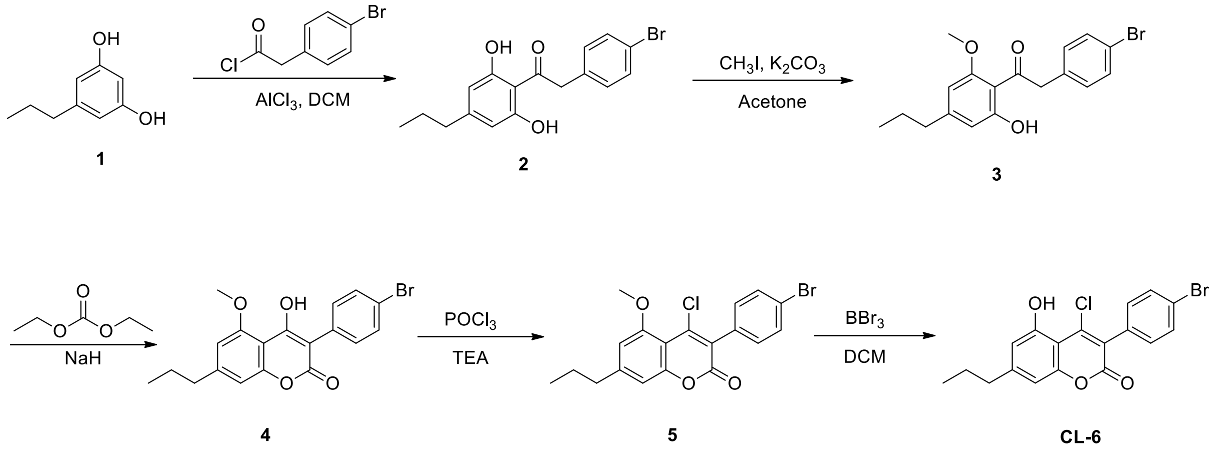

To a solution of **1** (6.75 g, 44.4 mmol), $AlCl_3$ (11.84 g, 88.8 mmol) in DCM stirred under nitrogen at 0 °C was added 2-(4-bromophenyl)acetyl chloride (10.37 g, 44.4 mmol) dropwise. The mixture was warmed to room temperature and stirred for 16 hours. The reaction mixture was quenched with 50 mL crushed ice and diluted with EtOAc. Then the mixture was washed with 1M HCl and saturated brine. The combined organic layer was dried over anhydrous Na_2SO_4 and concentrated in vacuum. The residue was purified by flash chromatography on silica gel (PE/EA = 8/1) to afford compound **2** (11.79 g, 68% yield) as a yellow solid. ^{1}H NMR (300 MHz, DMSO-*d*$_6$): δ 11.78(s, 2H), 7.52 (d, *J* = 8.1 Hz, 2H), 6.26 (s, 2H), 7.22 (d, *J* = 8.4 Hz, 2H), 4.41 (s, 2H), 2.45 (t, *J* = 7.2 Hz, 2H), 1.60-1.53 (m, 2H), 0.91 (t, *J* = 7.2 Hz, 3H). LC-MS: m/z 349.0 $[M+H]^+$.

2_HNMR

To a solution of **2** (200 mg, 0.57 mmol) and K_2CO_3 (79.15 mg, 0.57 mmol) in acetone stirred under nitrogen at 25 °C was added CH_3I (81.29 mg, 0.57 mmol) dropwise. The mixture was sealed in a tube and stirred at 58 °C for 8 hours. The reaction mixture was cooled to room temperature and filtered. The filtrate was concentrated and purified by flash chromatography on silica gel (PE/EA = 5:1) to give the product compound **3** (34 mg, 16.3% yield) as a yellow oil. ^{1}H NMR (300 MHz, $CDCl_3$) δ: 13.09 (s, 1H), 7.45 (d, J = 8.4 Hz, 2H), 7.08 (d, J = 8.4 Hz, 2H), 6.42 (s, 1H), 6.21 (s, 1H), 4.31 (s, 2H), 3.89 (s, 3H), 2.53 (t, J = 7.8 Hz, 2H), 1.69-1.60 (m, 2H), 0.95 (t, J = 7.2 Hz, 3H). LC-MS: m/z 363.0 $[M+H]^+$.

3_HNMR.pdf

A solution of compound **3** (1.42 g, 4 mmol) in diethyl carbonate (10 mL) was added to a suspension of sodium hydride (60% dispersion in mineral oil, 0.94 g, 39 mmol) in diethyl carbonate (10 mL) at 0 °C. The mixture was heated to 100 °C and stirred for 3 hours. The reaction mixture was cooled to 0 °C and then quenched by dropwise addition of water until effervescence stopped. The pH of reaction mixture was adjusted to 2 with 2N HCl and extracted with EA (50 mL x 3). The combined organic phase was dried over Na_2SO_4, filtered and concentrated. The residue was triturated with EA to give compound **4** (1.1 g, 59% yield) as a white solid. LC-MS: m/z 388.6 $[M+H]^+$.

4_LCMS

To a solution of compound **4** (110 mg, 0.28 mmol)) in $POCl_3$ (2 mL) was added Triethylamine (43.83 mg, 0.34 mmol) at 0 °C. The mixture was warmed to 50 °C and stirred for 5 hours. The reaction mixture was quenched with aqueous $NaHCO_3$ and extracted with EA (10 mL x 3). The combined organic phase was dried over Na_2SO_4, filtered and concentrated. The residue was purified by flash chromatography on silica gel (PE/EA = 50/1) to give compound **5** (98 mg, 85%) as a white solid. LC-MS: m/z 306.6 $[M+H]^+$.

5_LCMS

To a solution of compound **5** (98 mg, 0.24 mmol) in DCM was added a solution of tribromoborane (1.2 mmol, 2 mol/L in DCM) dropwise under nitrogen at -70 ^{o}C. The reaction mixture was stirred at -70 ^{o}C and slowly warm to room temperature, and it was continued to stir for 2 hours. Then the reaction mixture was quenched with water and extracted with EA (10 mL x 3). The combined organic phase was dried over Na_2SO_4, filtrated and concentrated. The residue was purified by prep-HPLC (0.1% NH_4HCO_3 in H_2O/MeCN) to give **CL-6** (30 mg, 30%) as a white solid. ^{1}H NMR (400 MHz, CD_3OD) δ: 7.63-7.60 (m, 2H), 7.32 - 7.26 (m, 2H), 6.89 (s, 1H), 6.72 (s, 1H), 2.68 (t, *J* = 7.6 Hz, 2H), 1.72-1.66 (m, 2H), 0.98 (t, *J* = 7.2 Hz, 3H). LC-MS: m/z 392.8 $[M+H]^+$.

CL6_LCMS CL6_HNMR

Synthesis of compounds W7-W10

Lifan Zeng, Ph. D
Chemical Genomics Core Facility,
Department of Biochemistry and Molecular Biology
School of Medicine, Indiana University
Room 1007, 635 Barnhill Dr.,
Indianapolis, IN, 46202

The LCMS analysis of the compounds was performed on Agilent 1290 LC - 6545 QTOF. Agilent ZORBAX Eclipse Plus C18 column (HD 2.1*50mm 1.8-Micron) was used for separation. 0.1% formic acid in water (v:v) as mobile phase A and 0.1% formic acid acetonitrile as mobile phase B (v:v) were used. The flow rate is 0.6 mL/min. The gradient starts as 5% B and holds for 0.5 minute, increased from 5% B to 95 %B in 4.5 minutes and holds for 0.5-minute, 95%B to 5%B in 0.5 minute and holds for 0.5 minute. NMR spectrum was collected on Bruker NMR. The chemical shifts are reported as ppm (δ) relative to the residual solvent peak.

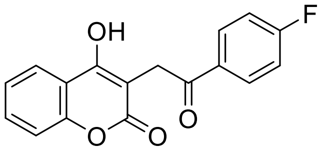

3-(2-(4-fluorophenyl)-2-oxoethyl)-4-hydroxy-2H-chromen-2-one (W7)

A mixture of 1-(4-fluorophenyl)-2,2-dihydroxyethan-1-one (0.1 mmol), 4-hydroxycoumarin (0.1 mmol) and 4-methoxythiophenol (0.1 mmol) in 1 mL of AcOH was heated to 130 degrees Celsius in microwave reactor for 30 min. The reaction mixture was cooled down to room temperature then poured into 15 mL of cold water. Precipitate was collected by filtration and purified by flash column chromatography using EtOAc and hexane as eluent to provide title compound as white solid. 1H NMR (300 MHz, DMSO-d6) δ 11.65 (bs, 1H), 8.19-8.16 (m, 2H), 7.96 (m, 1H), 7.64 (t, J = 10.1 Hz, 1H), 7.43-7.35 (m, 4H), 4.30 (s, 2H); Purity (98.5%, 254 nm); ESI positive mode, 299.1106 $[M+H]^+$.

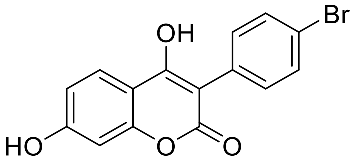

3-(4-bromophenyl)-4,7-dihydroxy-2H-chromen-2-one (W8)

W8 is an in-house compound from Chemical Genomics Core. 1H NMR (300 MHz, DMSO-d6) δ 10.59 (s, 1H), 7.85 (m, 2H), 7.58 (m, 1H), 7.34 (m, 2H), 6.80 (m, 1H), 6.71 (s, 1H); Purity (98.7%, 334 nm); ESI positive mode, 334.9752 $[M+H]^+$.

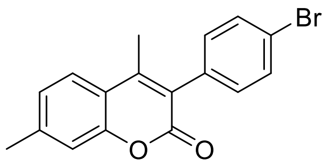

3-(4-bromophenyl)-4,7-dimethyl-2H-chromen-2-one (W9)

W8 is an in-house compound from Chemical Genomics Core. 1H NMR (300 MHz, DMSO-d6) δ 7.76 (m, 1H), 7.64 (m, 2H), 7.31-7.23 (m, 4H), 2.44 (s, 3H), 2.25 (s, 3H); Purity (100%, 274 nm); ESI positive mode, 329.0156 $[M+H]^+$.

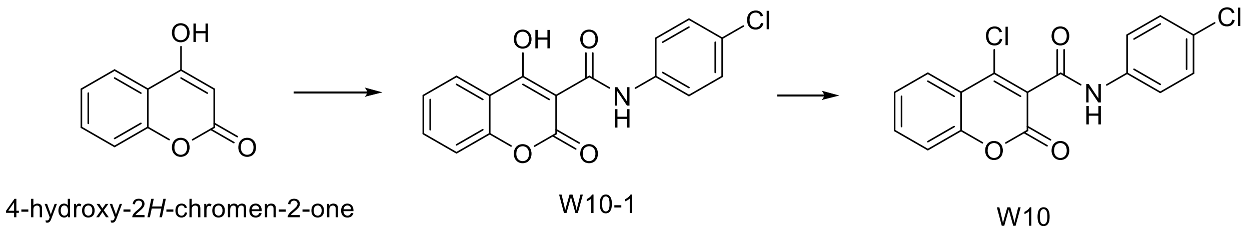

4-chloro-N-(4-chlorophenyl)-2-oxo-2H-chromene-3-carboxamide (W10)

20 mmol of 4-hydroxy-2H-chromen-2-one was dissolved in 30ml of dry DMSO, to which 20 mmol of pyridine and 20 mmol of 4-chlorophenyl isocyanate were added in one portion. The mixture was stirred under N2 overnight at room temperature. After adding 100 ml of 1N HCl, the mixture was stirred for 10min and the precipitate was filtered, and washed with water, EtOH, and ether. The solid was dried in vacuo overnight to give 1.14g of W10-1. 500mg of W10-1 was suspended in 20 ml of dry DCM with 3 drops of DMF. 15 mmol of oxalyl chloride was added dropwise at room temperature. The mixture was stirred overnight, and the solution became clear the next morning. The organic layer dried and evaporated to give white solid, which was purified by chromatography using DCM/Hexane to title compound as white solid. 1H NMR (300 MHz, DMSO-d6) δ 10.38 (s, 1H), 8.01 (m, 1H), 7.85 (m, 1H), 7.60 (m, 2H), 7.58 (m, 2H), 7.43 (m, 2H); Purity (100%, 254 nm); ESI positive mode, 334.0007 $[M+H]^+$.

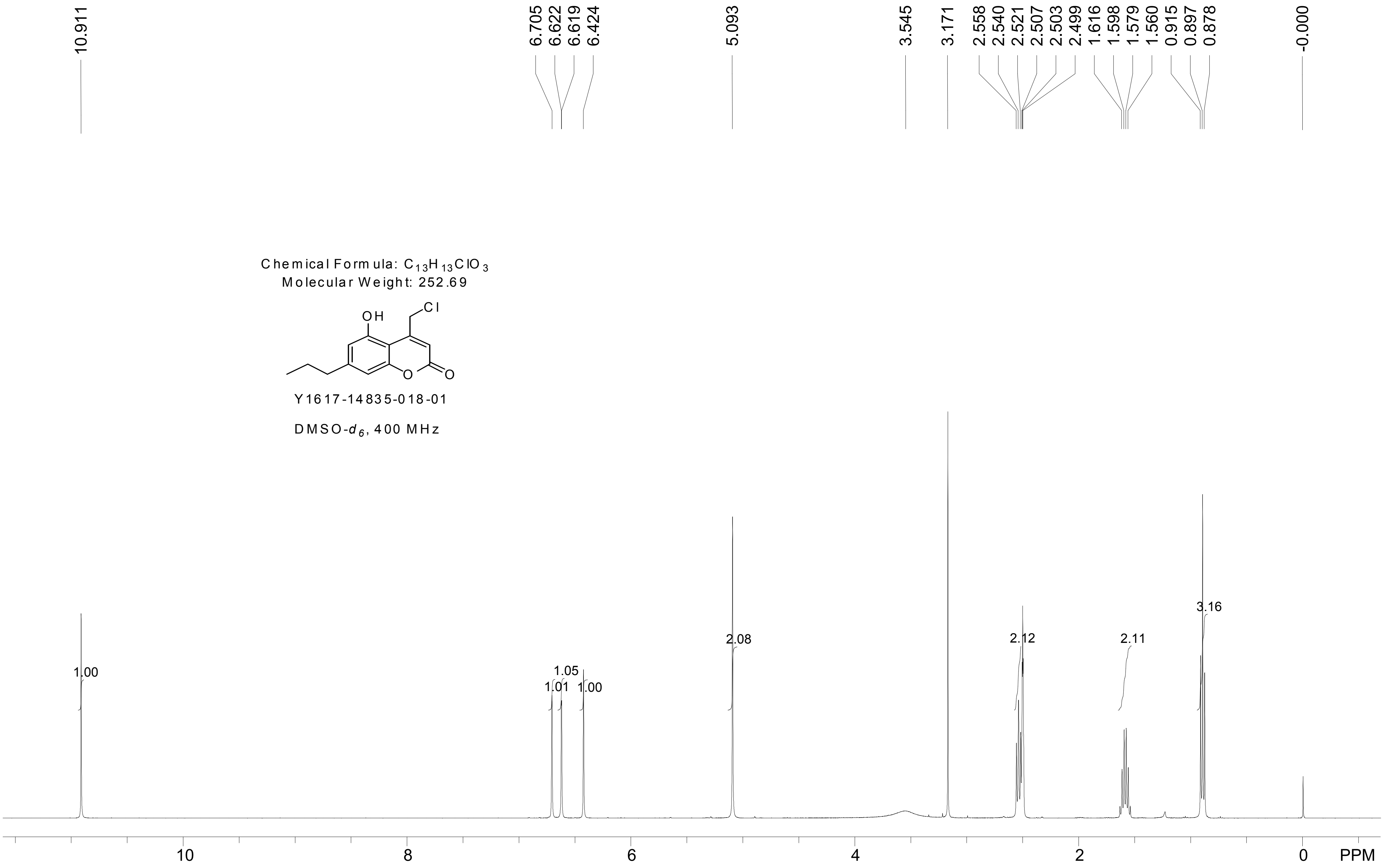
10.911
6.705
6.622
6.619
6.424
5.093
3.545
3.171
2.558
2.540
2.521
2.507
2.503
2.499
1.616
1.598
1.579
1.560
0.915
0.897
0.878
-0.000
Chemical Formula: $C_{13}H_{13}ClO_3$
Molecular Weight: 252.69
OH
Cl
O
O
Y1617-14835-018-01
DMSO-d_6, 400 MHz
1.00
1.01
1.05
1.00
2.08
2.12
2.11
3.16
10
8
6
4
2
0
PPM

388 Jialilue Road
Zhangjiang Hightech
Shanghai, China
Post code : 201203
Tel : 8621-51098642
Fax : 8621-51903505

Sundia Meditech Co.Ltd

QC Report

Sample Name	: Y1617-14835-018-01		
Data File	: D:\DATA-LCMS-5\2020\03-20\05\1\Y1617-14835-018-01-.D		
Injection Date	: Thu, 5. Mar. 2020	Inj. Vol.	: 2ul
Acq Operator	: lcms-5	Location	: Vial 32
Acq. Method	: D:\data-lcms-5\2020\03-20\05\1\P-20-95-TFA.M		
Sample Info.	: column: ZORBAX C18 5um 4.6*50mm ; mobile phase:B(ACN), A(0.05%TFA); gradient(B%):as Acq.Method above		

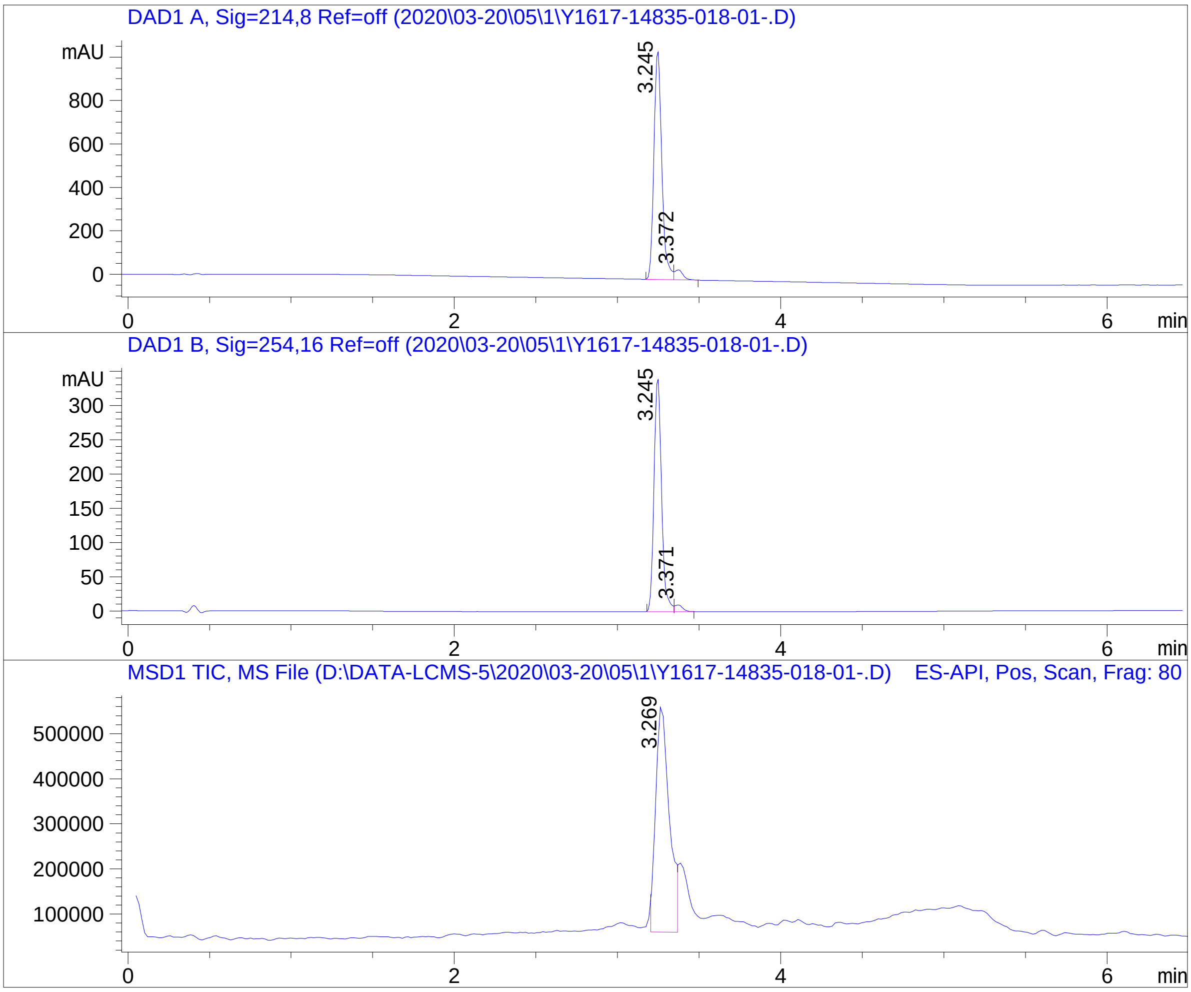

Integration Results

Signal 1: DAD1 A, Sig=214,8 Ref=off

#	R.T.	Type	Height	Height%	Width	Area	Area %
1	3.245	BV	1051.217	95.823	0.051	3538.250	95.617
2	3.372	VB	45.820	4.177	0.053	162.172	4.383

Signal 2: DAD1 B, Sig=254,16 Ref=off

#	R.T.	Type	Height	Height%	Width	Area	Area %
1	3.245	BV	340.364	97.212	0.050	1096.499	97.060
2	3.371	VB	9.763	2.788	0.052	33.213	2.940

Signal 3: MSD1 TIC, MS File

#	R.T.	Type	Height	Height%	Width	Area	Area %
1	3.269	FM	511211.750	100.000	0.095	2.912e+006	100.000

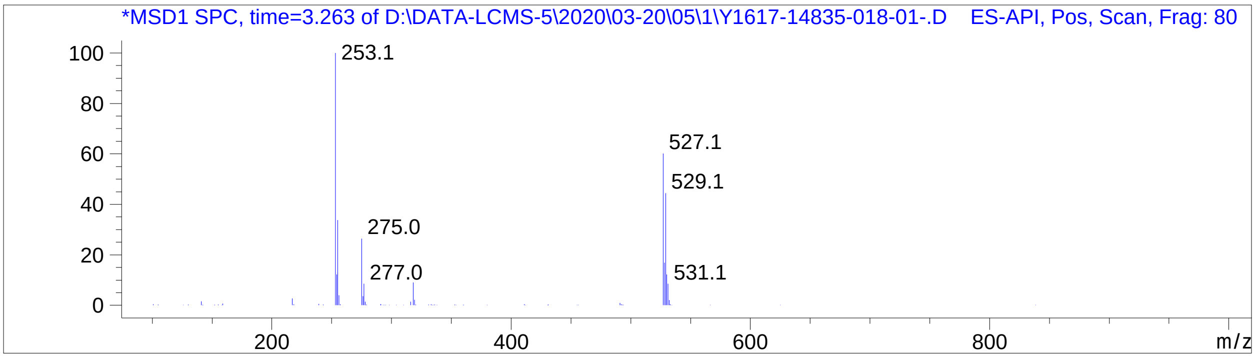

==

*** End of Report ***

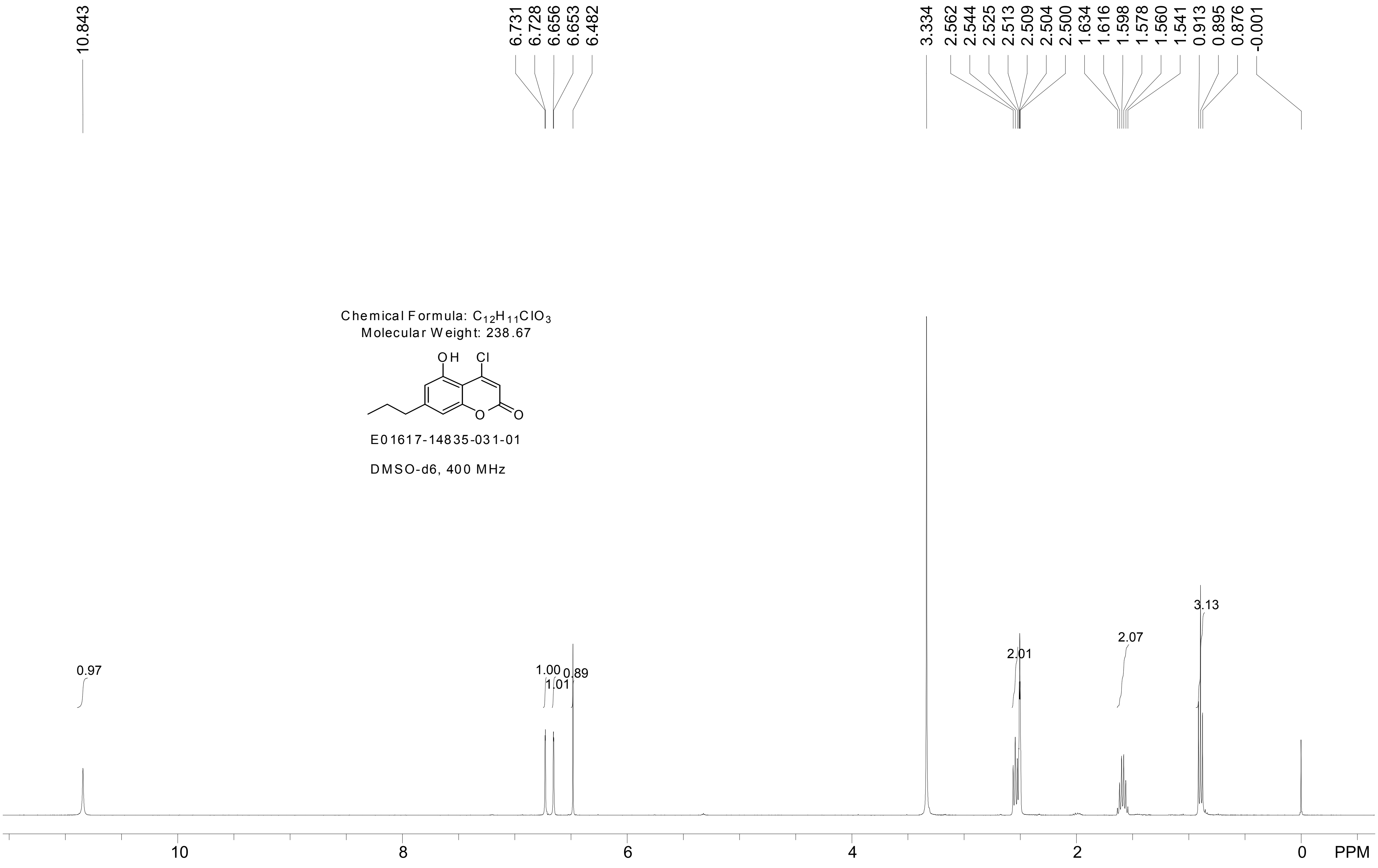

10.843
6.731
6.728
6.656
6.653
6.482
3.334
2.562
2.544
2.525
2.513
2.509
2.504
2.500
1.634
1.616
1.598
1.578
1.560
1.541
0.913
0.895
0.876
-0.001
Chemical Formula: $C_{12}H_{11}ClO_3$
Molecular Weight: 238.67
OH
Cl
O
O
E01617-14835-031-01
DMSO-d6, 400 MHz
0.97
1.00
1.01
0.89
2.01
2.07
3.13
10
8
6
4
2
0
PPM

Sundia Meditech Co.Ltd

388 Jialilue Road
Zhangjiang Hightech Park
Shanghai, China
Post code : 201203
Tel : 8621-51098642
Fax : 8621-51903505

QC Report

Sample Name : E01617-14835-031-01
Data File : D:\DATA-LCMS-5\2020\06-20\15\1\E01617-14835-031-01-.D
Injection Date : Mon, 15. Jun. 2020 Inj. Vol. : 5ul
Acq Operator : lcms-5 Location : Vial 5
Acq. Method : D:\data-lcms-5\2020\06-20\15\1\P-30-70-TFA.M
Method Info. : column:EC C18 4um 4.6*50mm ;mobile phase:B(ACN), A(0.05% TFA); gradient(B%):as Acq.Method above

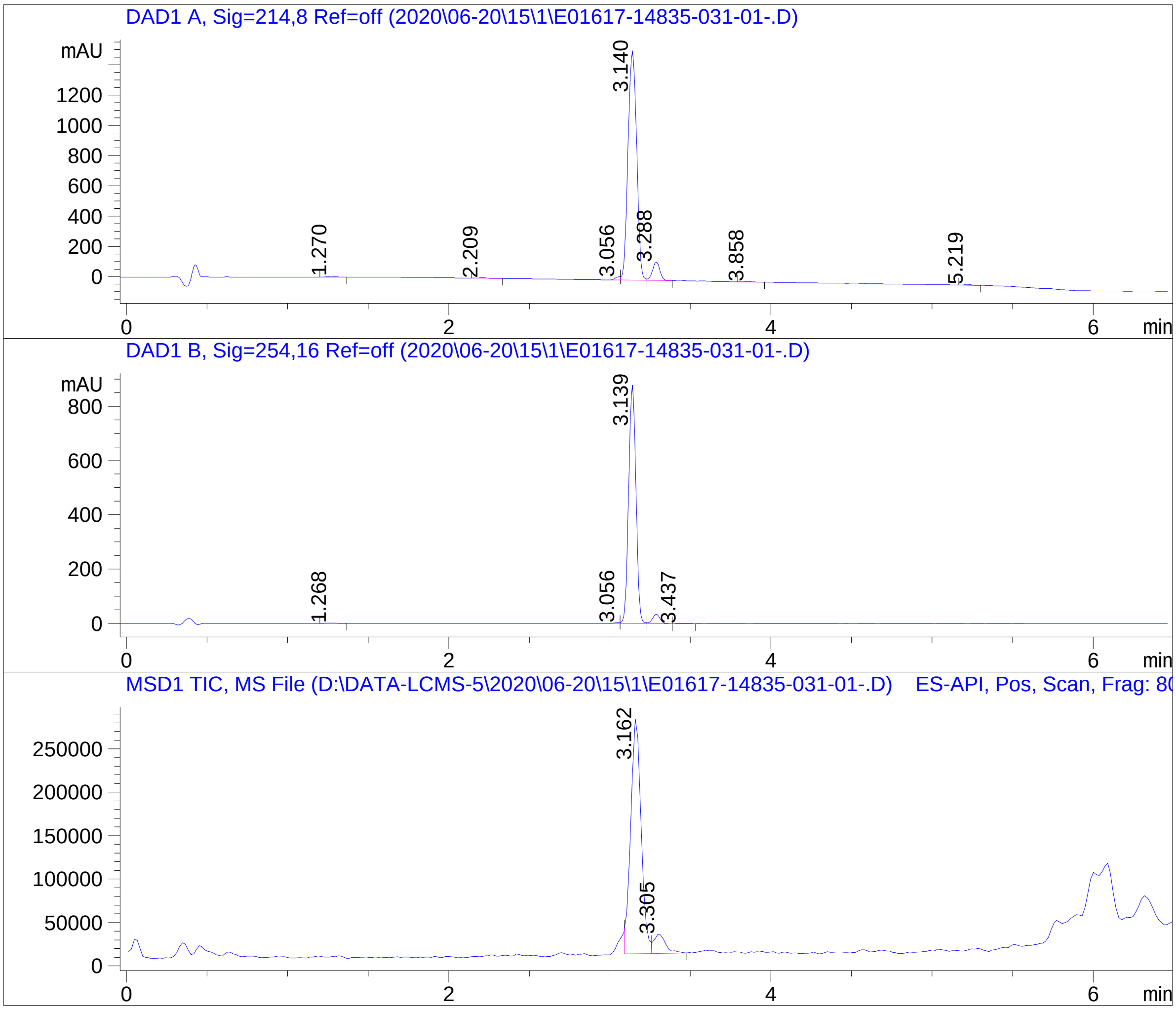

===

Integration Results

===

Signal 1: DAD1 A, Sig=214,8 Ref=off

#	R.T.	Type	Height	Height%	Width	Area	Area %
1	1.270	BB	4.814	0.288	0.053	16.046	0.261
2	2.209	BB	3.147	0.188	0.064	12.539	0.204
3	3.056	MF	20.668	1.235	0.038	47.627	0.775
4	3.140	FM	1514.989	90.548	0.062	5654.296	91.972
5	3.288	VB	121.377	7.254	0.051	388.959	6.327
6	3.858	BB	2.885	0.172	0.047	8.708	0.142
7	5.219	BB	5.261	0.314	0.056	19.693	0.320

#	R.T.	Type	Height	Height%	Width	Area	Area %

Signal 2: DAD1 B, Sig=254,16 Ref=off

#	R.T.	Type	Height	Height%	Width	Area	Area %
1	1.268	BB	1.345	0.146	0.059	5.214	0.180
2	3.056	MF	4.217	0.457	0.039	9.779	0.337
3	3.139	FM	881.458	95.594	0.052	2773.314	95.558
4	3.288	VB	34.041	3.692	0.052	110.031	3.791
5	3.437	BB	1.024	0.111	0.058	3.889	0.134

Signal 3: MSD1 TIC, MS File

#	R.T.	Type	Height	Height%	Width	Area	Area %
1	3.162	FM	276712.188	92.543	0.074	1.229e+006	91.535
2	3.305	VB	22297.127	7.457	0.085	113608.313	8.465

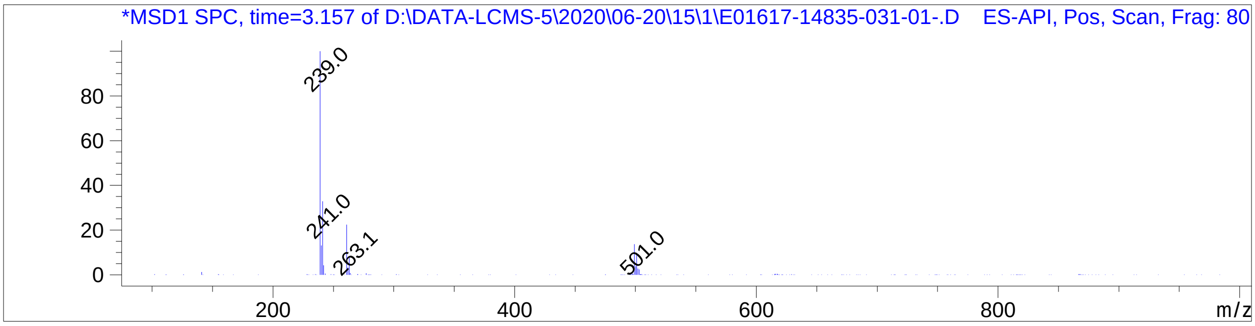

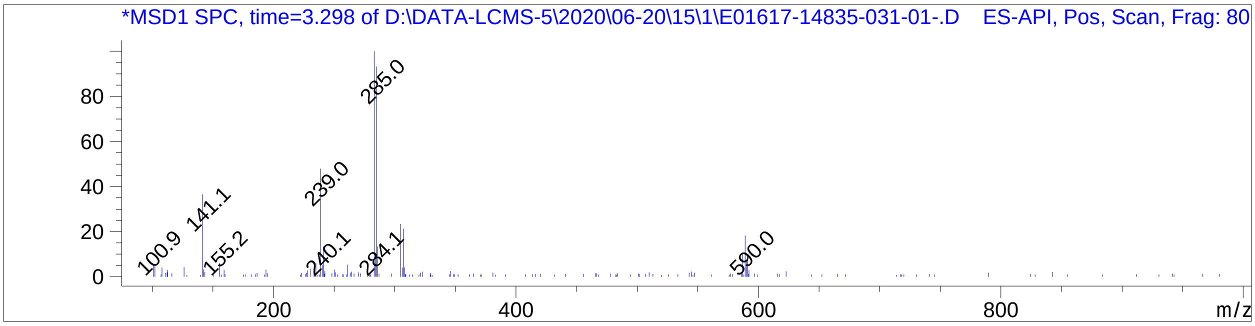

*** End of Report ***

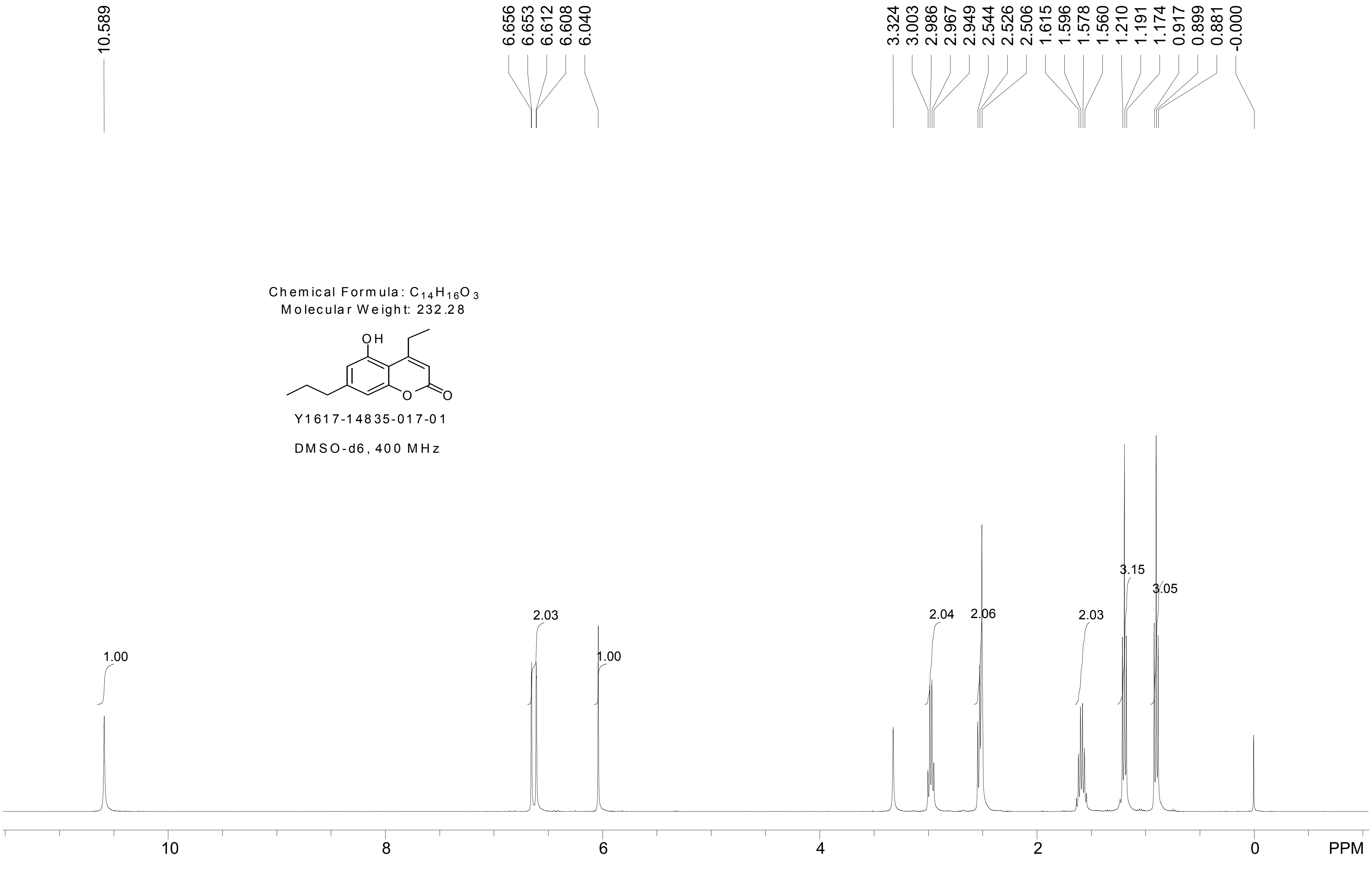

Chemical Formula: $C_{14}H_{16}O_3$
Molecular Weight: 232.28

Y1617-14835-017-01

DMSO-d6, 400 MHz

Sundia Meditech Co.Ltd

QC Report

388 Jialilue Road
Zhangjiang Hightech Park
Shanghai, China
Post code : 201203
Tel : 8621-51098642
Fax : 8621-51903505

Sample Name	: Y1617-14835-017-01			
Data File	: D:\DATA-LCMS-13\2020\03-20\06\2\Y1617-14835-017-01.D			
Injection Date	: Fri, 6. Mar. 2020	Inj. Vol.	:	3ul
Acq Operator	: LCMS-13	Location	:	Vial 91
Acq. Method	: D:\data-lcms-13\2020\03-20\06\2\P-05-95.M			
Method Info.	: column:Ecllpse XDB-C18 5um 4.6*150mm ;mobile phase:B(ACN), A(0.02%NH4AC); gradient(B%):as Acq.Method above			

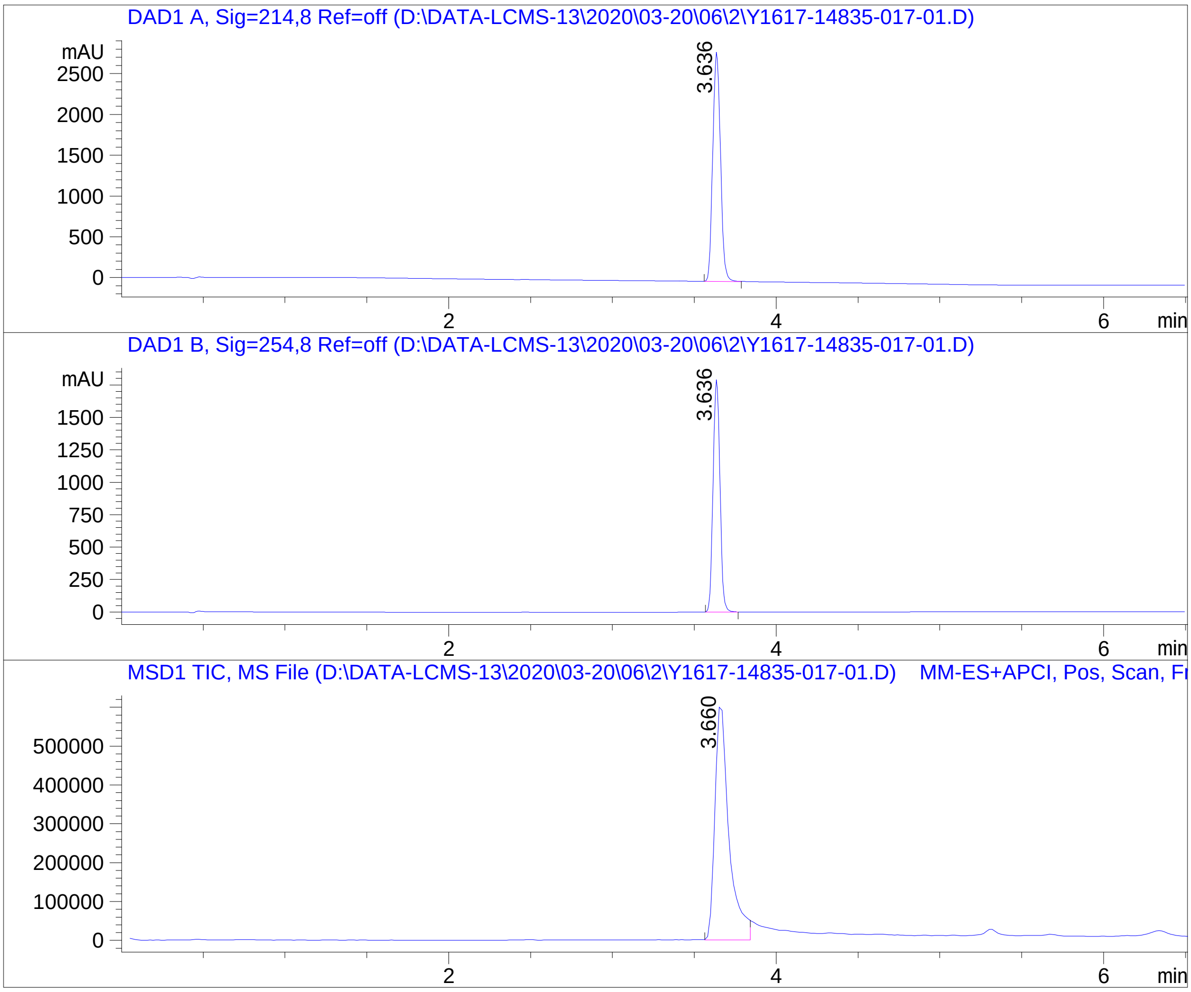

==

Integration Results

==

Signal 1: DAD1 A, Sig=214,8 Ref=off

#	R.T.	Type	Height	Height%	Width	Area	Area %
1	3.636	BB	2816.121	100.000	0.052	9264.238	100.000

Signal 2: DAD1 B, Sig=254,8 Ref=off

#	R.T.	Type	Height	Height%	Width	Area	Area %
1	3.636	BB	1794.671	100.000	0.046	5261.366	100.000

Signal 3: MSD1 TIC, MS File

#	R.T.	Type	Height	Height%	Width	Area	Area %
1	3.660	MM	621897.625	100.000	0.097	3.633e+006	100.000

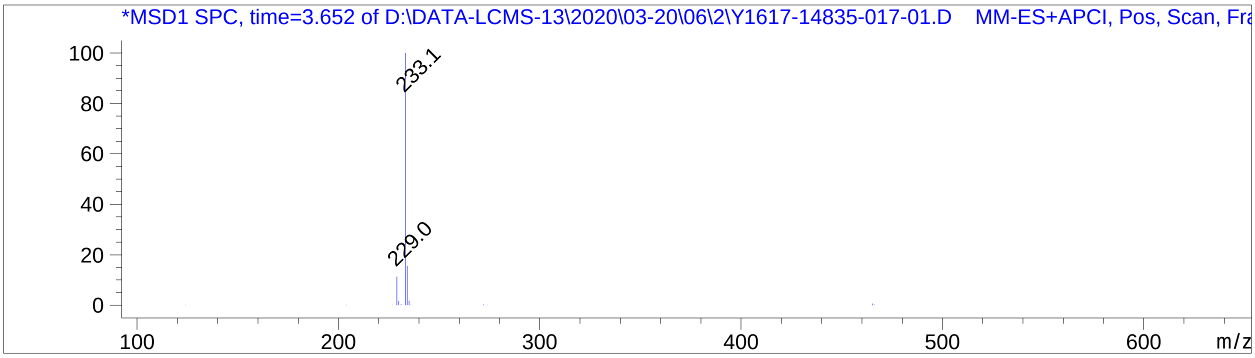

*** End of Report ***

Sundia Meditech Co.Ltd

QC Report

388 Jialilue Road
Zhangjiang Hightech Par
Shanghai, China
Post code : 201203
Tel : 8621-51098642
Fax : 8621-51903505

Sample Name : E03594-ENB227306-044-01
Data File : D:\DATA-LCMS5\2022\202211\25\1\E03594-ENB227306-044-01.D
Injection Date : Fri, 25. Nov. 2022 Inj. Vol. : 2ul
Acq Operator : lcms-5 Location : Vial 24
Acq. Method : D:\data-lcms5\2022\202211\25\1\C2-P-70-95-15MIN-TF
Sample Info.: column: ZORBAX SB-C8 ;column size: 4.6*150mm,3.5um ; mobile phase:B(ACN),A(0.05%TFA); gradient(B%):as Acq. Method above

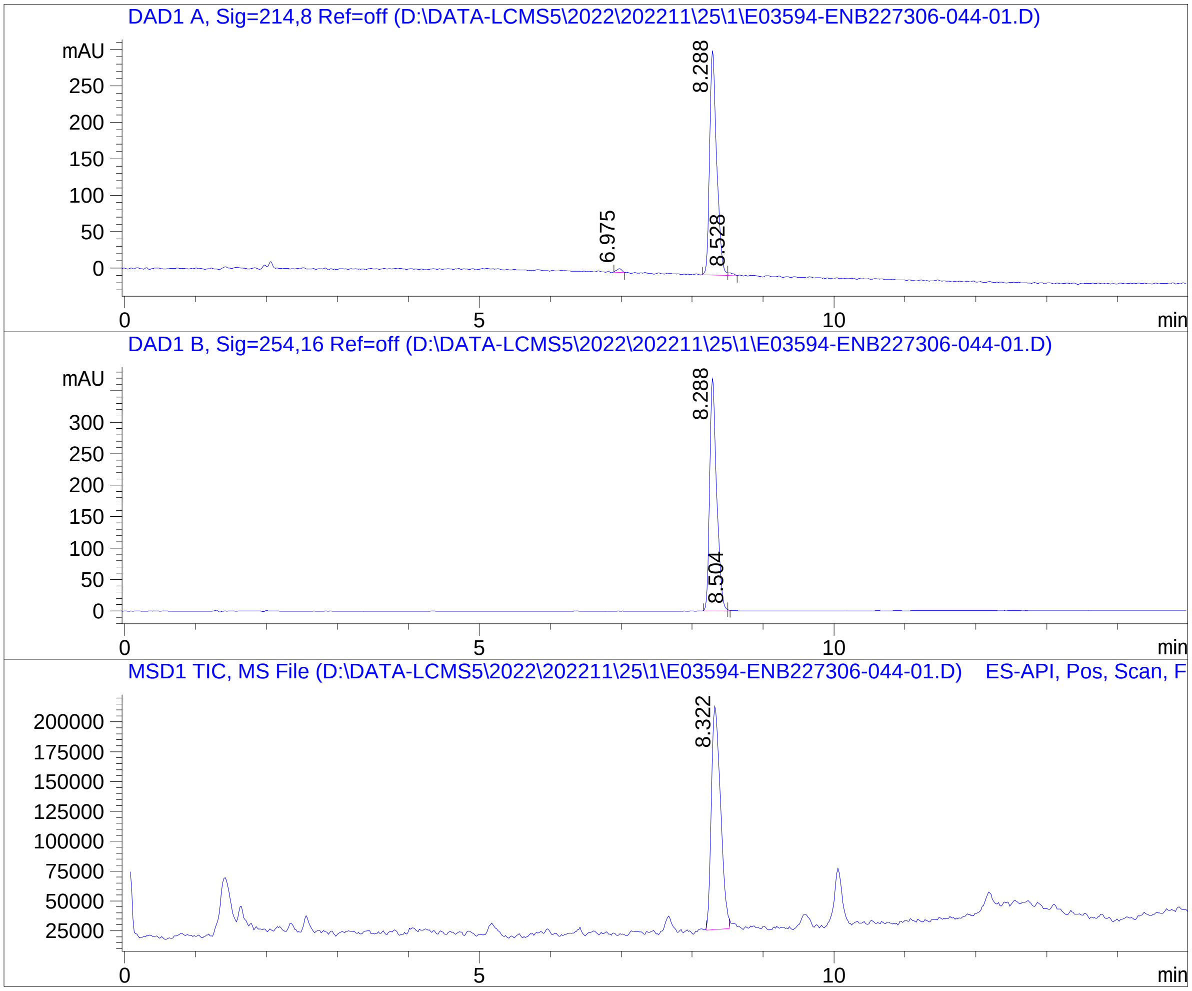

Integration Results

Signal 1: DAD1 A, Sig=214,8 Ref=off

#	R.T.	Type	Height	Height%	Width	Area	Area %
1	6.975	MM	5.041	1.594	0.083	25.183	1.167
2	8.288	MF	307.394	97.233	0.115	2114.688	98.017
3	8.528	FM	3.707	1.173	0.079	17.603	0.816

#	R.T.	Type	Height	Height%	Width	Area	Area %

Signal 2: DAD1 B, Sig=254,16 Ref=off

#	R.T.	Type	Height	Height%	Width	Area	Area %
1	8.288	MF	369.577	99.548	0.114	2517.349	99.919
2	8.504	FM	1.678	0.452	0.020	2.046	0.081

Signal 3: MSD1 TIC, MS File

#	R.T.	Type	Height	Height%	Width	Area	Area %
1	8.322	MF	188447.359	100.000	0.150	1.692e+006	100.000

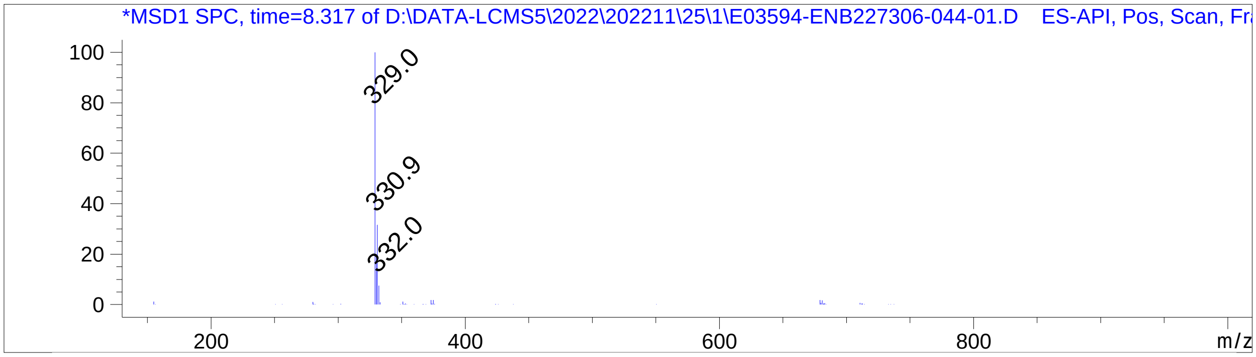

*** End of Report ***

Bruker Avance III 400 MHZ
Solvent: CD_3OD
Indiana

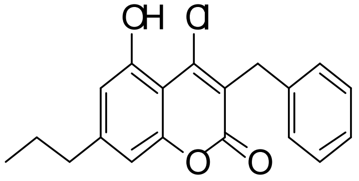

Chemical Formula: $C_{19}H_{17}ClO_3$
Exact Mass: 328.09
Molecular Weight: 328.79

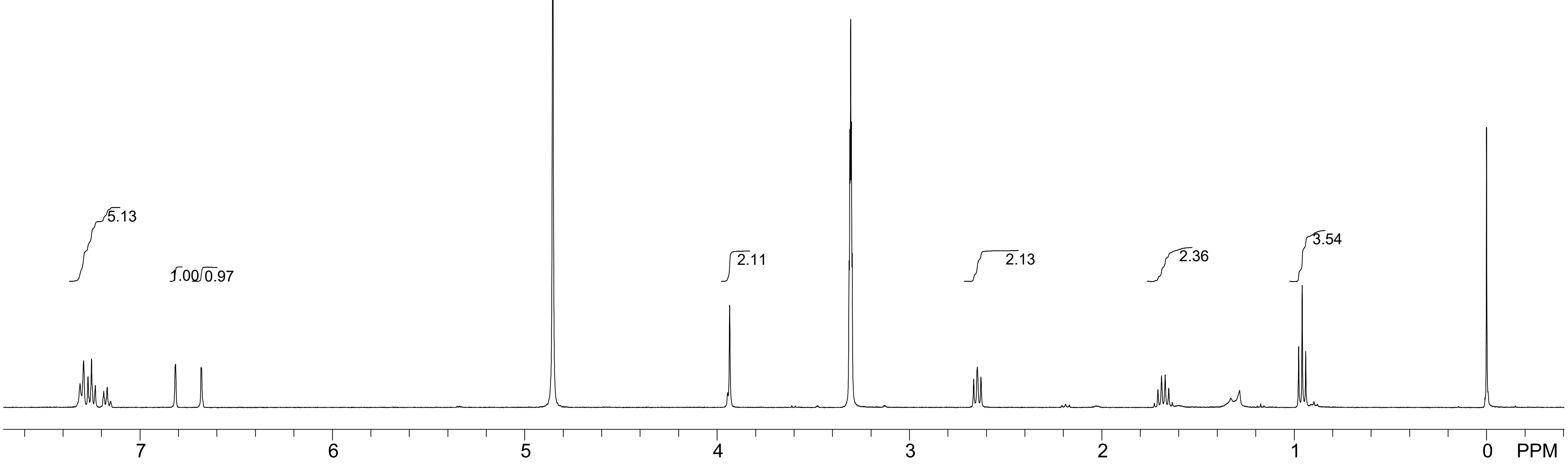

Sundia Meditech Co.Ltd

QC Report

388 Jialilue Road
Zhangjiang Hightech Park
Shanghai , China
Post code :201203
Tel : 8621-51098642
Fax : 8621-50790032

Sample Name	: E03594-ENB227306-060-01
Data File	: D:\data-RPHPLC2\2022\202212\13\E03594-ENB227306-060-01----.lcd
Injection Date	: 12/13/2022
Inj. Vol.	: 0.2 ul
Vial#	: 19
Acq. Operator	: LCMS-26
Acq. Method	: D:\methods\A\1.2ml\A-6.5min-70-95.lcm
Oven Temperature	: 40 C
Column	: Waters Sunfire C18 3.5um, 50*4.6mm
Mobile Phase A	: 0.02%NH4AC aq.
Mobile Phase B	: ACN
Total Flow	: 1.2ml/min

Chromatogram

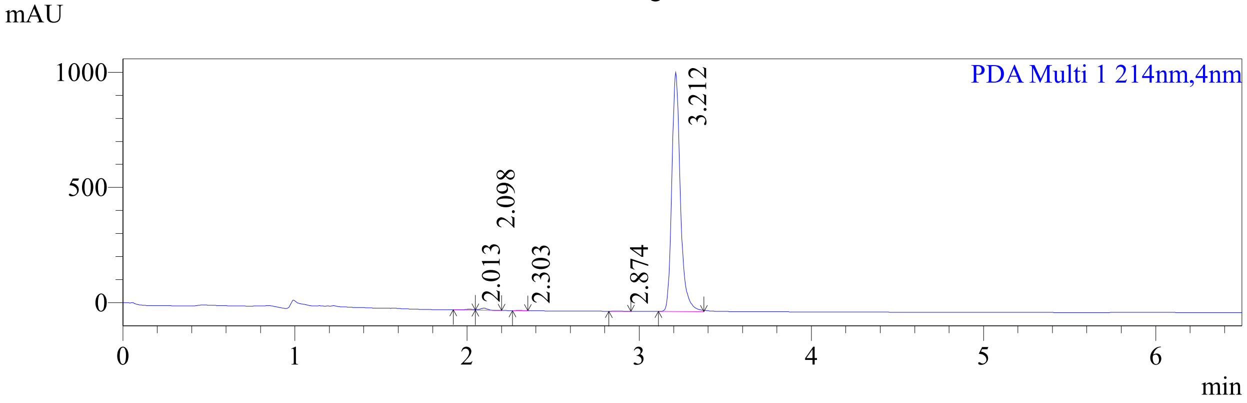

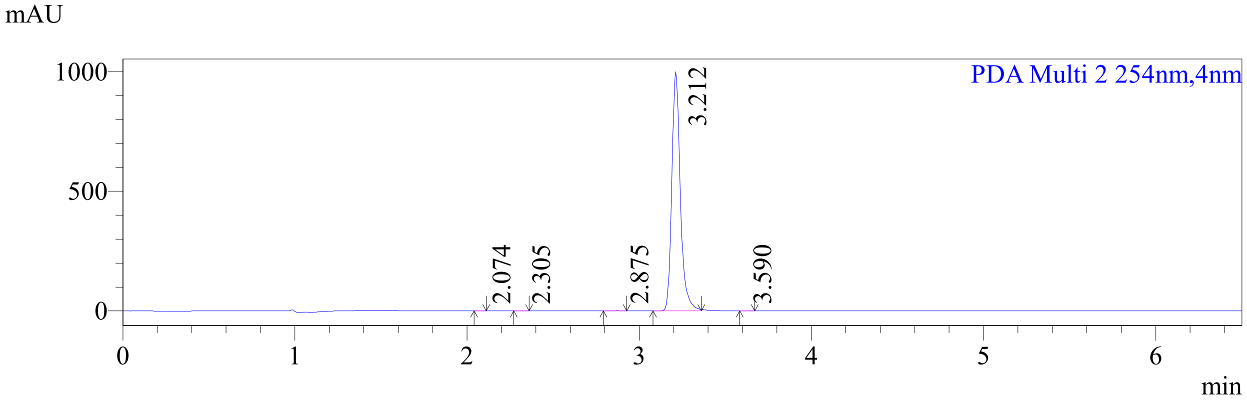

Peak Table

PDA Ch1 214nm

#	R.T.	Mark	Height	Area	Area%	Tailing Factor	Resolution
1	2.013		4206.08	14440.14	0.40	--	--
2	2.098	V	9437.64	29388.09	0.81	--	0.91
3	2.303		1003.07	2392.05	0.07	1.08	2.57
4	2.874	M	1036.17	3388.98	0.09	1.39	7.03
5	3.212	M	1027698.02	3589440.28	98.64	1.28	3.69

PDA Ch2 254nm

#	R.T.	Mark	Height	Area	Area%	Tailing Factor	Resolution
1	2.074	M	498.30	1096.14	0.03	1.04	--
2	2.305	M	566.27	1395.98	0.04	1.16	3.42
3	2.875	M	1198.17	3896.56	0.11	--	7.02

#	R.T.	Mark	Height	Area	Area%	Tailing Factor	Resolution
4	3.212	M	986949.87	3448762.97	99.77	1.28	3.65
5	3.590	M	373.17	1531.04	0.04	--	0.20

Sundia Meditech Co.Ltd
QC Report

388 Jialilue Road
Zhangjiang Hightech Park
Shanghai, China
Post code : 201203
Tel : 8621-51098642
Fax : 8621-51903505

```
Sample Name     : E03594-ENB227306-060-01
Data File       : D:\DATA-LCMS13\2022\202212\13\1\E03594-ENB227306-060-0->
Injection Date  : Tue, 13. Dec. 2022             Inj. Vol.  : 0.30000001ul
Acq Operator    : LCMS-13                        Location   : Vial 41
Acq. Method     : D:\data-lcms13\2022\202212\13\1\P-70-95-2ML-TFA.M
Method Info.    : column:Diamonsil Plus,5um 4.6*30mm ;mobile phase:B(ACN),
                  A(0.05%TFA aq.); gradient(B%):as Acq.Method above
```

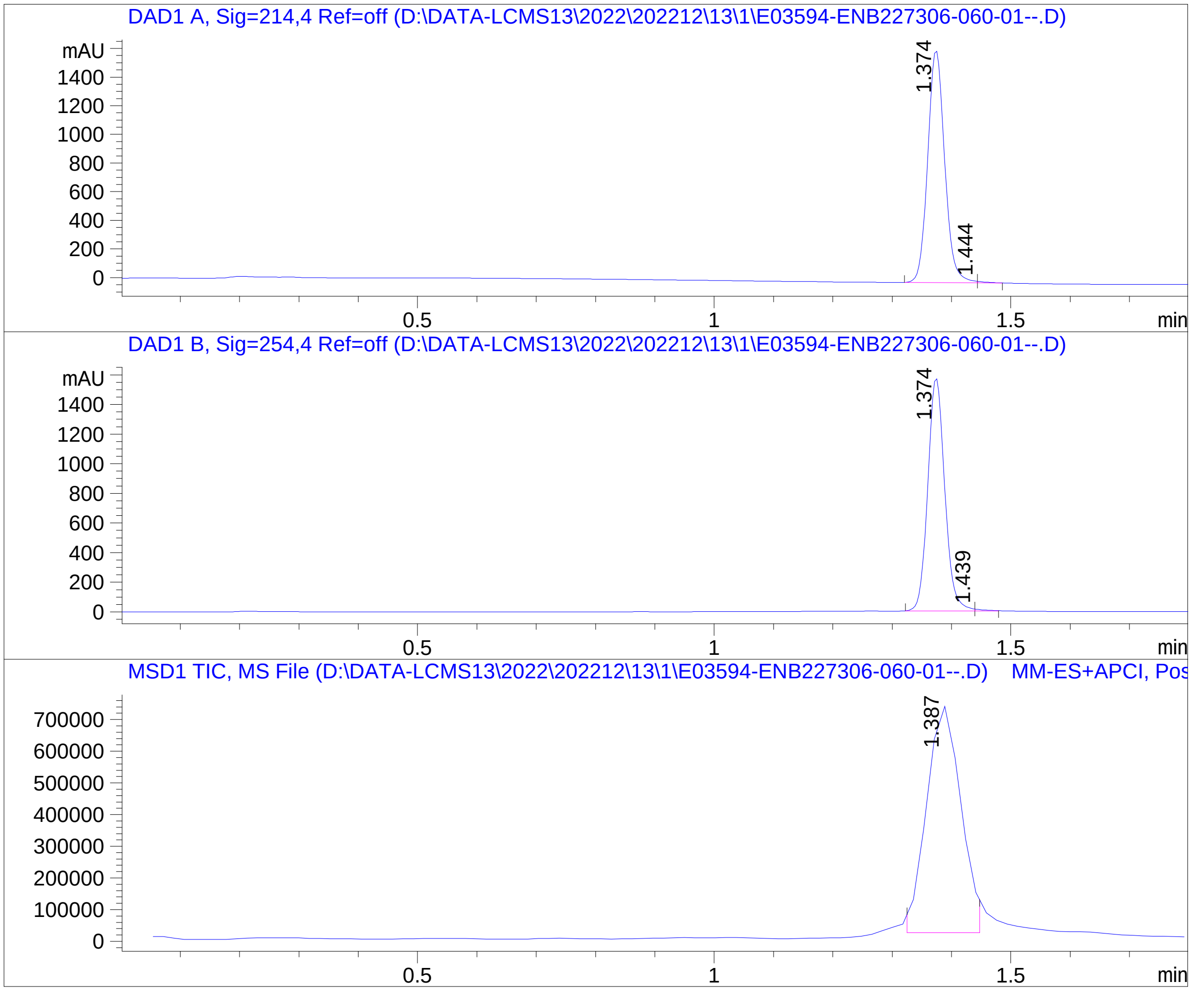

===

Integration Results

===

Signal 1: DAD1 A, Sig=214,4 Ref=off

#	R.T.	Type	Height	Height%	Width	Area	Area %
1	1.374	MF	1627.19	99.35	0.03	3219.91	99.68
2	1.444	FM	10.60	0.65	0.02	10.26	0.32

Signal 2: DAD1 B, Sig=254,4 Ref=off

#	R.T.	Type	Height	Height%	Width	Area	Area %
1	1.374	MF	1576.79	99.23	0.03	3126.26	99.61
2	1.439	FM	12.26	0.77	0.02	12.14	0.39

Signal 3: MSD1 TIC, MS File

#	R.T.	Type	Height	Height%	Width	Area	Area %
1	1.387	MM	727398.44	100.00	0.07	2839274.00	100.00

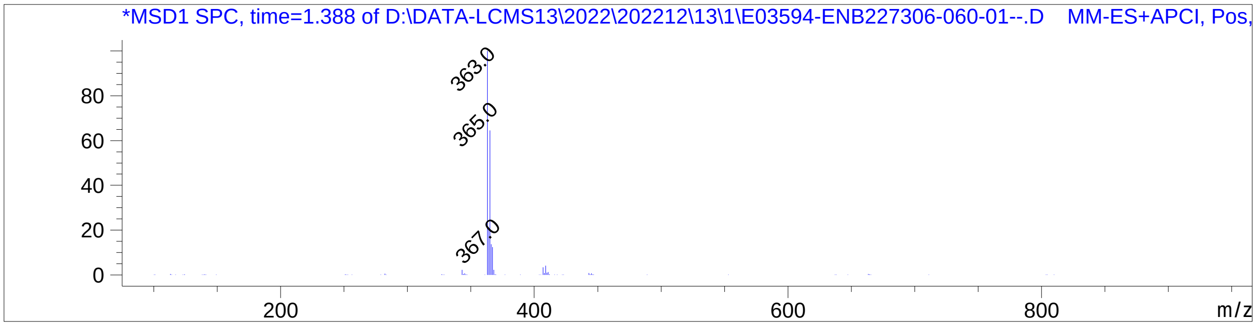

==
*** End of Report ***

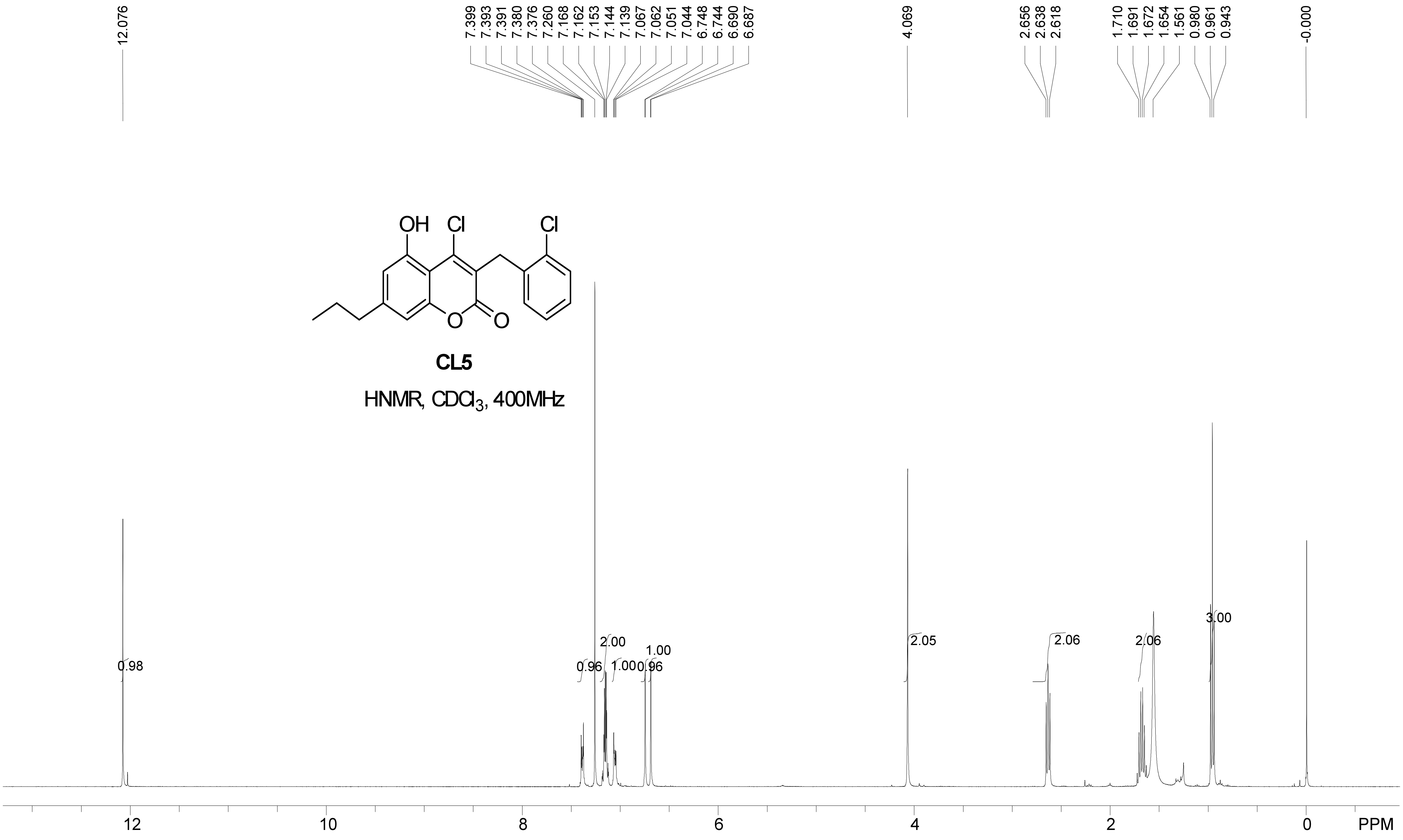
12.076
7.399
7.393
7.391
7.380
7.376
7.260
7.168
7.162
7.153
7.144
7.139
7.067
7.062
7.051
7.044
6.748
6.744
6.690
6.687
4.069
2.656
2.638
2.618
1.710
1.691
1.672
1.654
1.561
0.980
0.961
0.943
-0.000
OH
Cl
Cl
O
O
CL5
HNMR, CDCl3, 400MHz
0.98
0.96
2.00
1.00
1.00
0.96
2.05
2.06
2.06
3.00
12
10
8
6
4
2
0
PPM

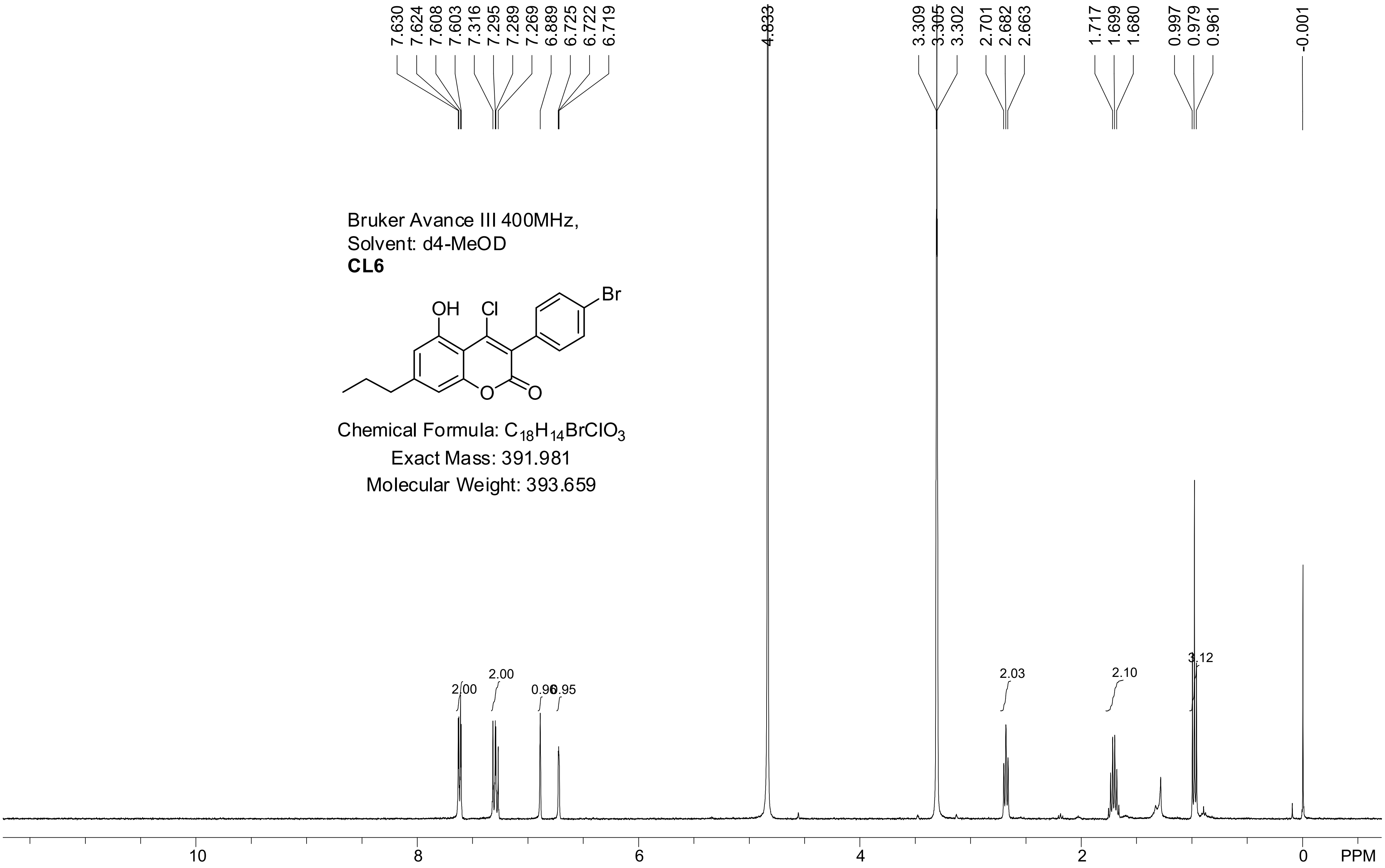
7.630
7.624
7.608
7.603
7.316
7.295
7.289
7.269
6.889
6.725
6.722
6.719
4.833
3.309
3.305
3.302
2.701
2.682
2.663
1.717
1.699
1.680
0.997
0.979
0.961
-0.001
Bruker Avance III 400MHz,
Solvent: d4-MeOD
CL6
OH
Cl
Br
O
O
Chemical Formula: C18H14BrClO3
Exact Mass: 391.981
Molecular Weight: 393.659
2.00
2.00
0.96
0.95
2.03
2.10
3.12
10
8
6
4
2
0
PPM

Sundia Meditech Co.Ltd
QC Report

388 Jialilue Road
Zhangjiang Hightech Par
Shanghai, China
Post code : 201203
Tel : 8621-51098642
Fax : 8621-51903505

Sample Name : E03722-ENB227271-047-001
Data File : D:\DATA-LCMS5\2022\202211\25\1\E03722-ENB227271-047-00->
Injection Date : Fri, 25. Nov. 2022   Inj. Vol. : 2ul
Acq Operator : lcms-5   Location : Vial 23
Acq. Method : D:\data-lcms5\2022\202211\25\1\C2-P-70-95-15MIN-TF
Sample Info.: column: ZORBAX SB-C8 ;column size: 4.6*150mm,3.5um ; mobile phase:B(ACN),A(0.05%TFA); gradient(B%):as Acq. Method above

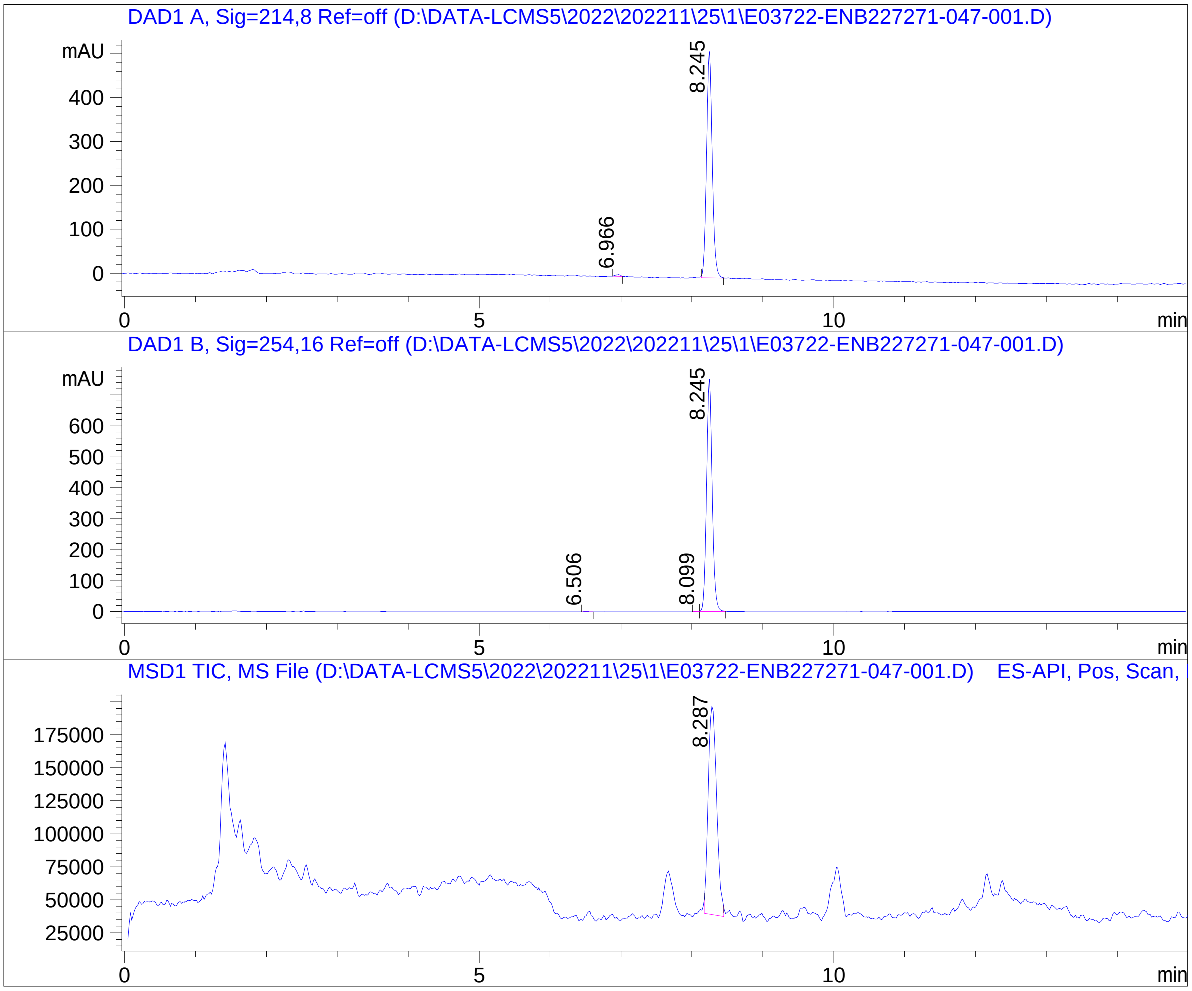

==
Integration Results
==

Signal 1: DAD1 A, Sig=214,8 Ref=off

#	R.T.	Type	Height	Height%	Width	Area	Area %
1	6.966	MM	3.710	0.713	0.081	17.998	0.614
2	8.245	BB	516.318	99.287	0.088	2911.518	99.386

Signal 2: DAD1 B, Sig=254,16 Ref=off

#	R.T.	Type	Height	Height%	Width	Area	Area %
1	6.506	MM	1.008	0.133	0.096	5.811	0.138
2	8.099	MF	1.473	0.195	0.051	4.527	0.108
3	8.245	FM	753.035	99.672	0.093	4192.384	99.754

Signal 3: MSD1 TIC, MS File

#	R.T.	Type	Height	Height%	Width	Area	Area %
1	8.287	MM	158636.406	100.000	0.137	1.303e+006	100.000

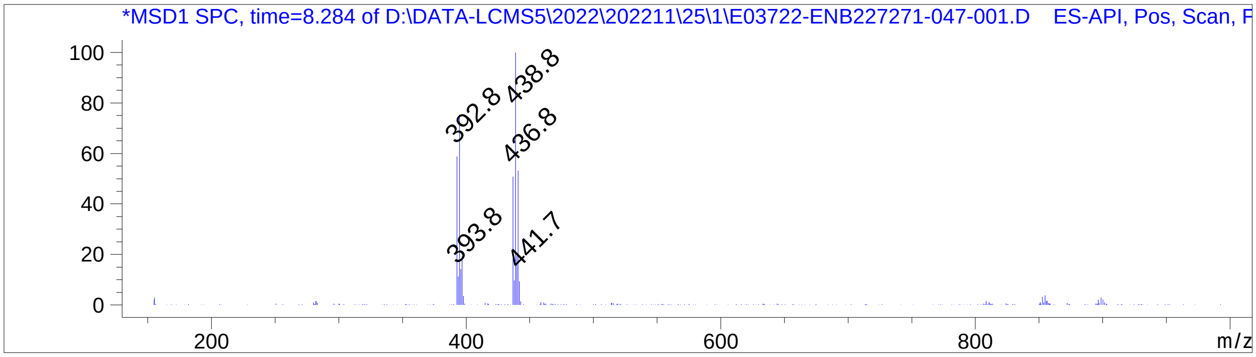

==

*** End of Report ***

Qualitative Analysis Report

**Data File**	20220824 ZL1 20 ul.d	**Sample Name**	
**Sample Type**	Sample	**Position**	P2-F1
**Instrument Name**	LC QTOF	**User Name**	
**Acq Method**	Small Molecule Training_0.6mLPerMin_7Min.m	**Acquired Time**	8/24/2022 2:26:09 PM (UTC-04:00)
**IRM Calibration Status**	Success	**DA Method**	default.m
**Comment**			
**Sample Group**		**Info.**	
**Stream Name**	LC 1	**Acquisition Time (Local)**	8/24/2022 2:26:09 PM (UTC-04:00)
**Acquisition SW Version**	6200 series TOF/6500 series Q-TOF B.08.00 (B8058.0)	**QTOF Driver Version**	8.00.00
**QTOF Firmware Version**	20.698	**Tune Mass Range Max.**	3200

Chromatograms

Fragmentor Voltage 220 **Collision Energy** 0 **Ionization Mode** ESI

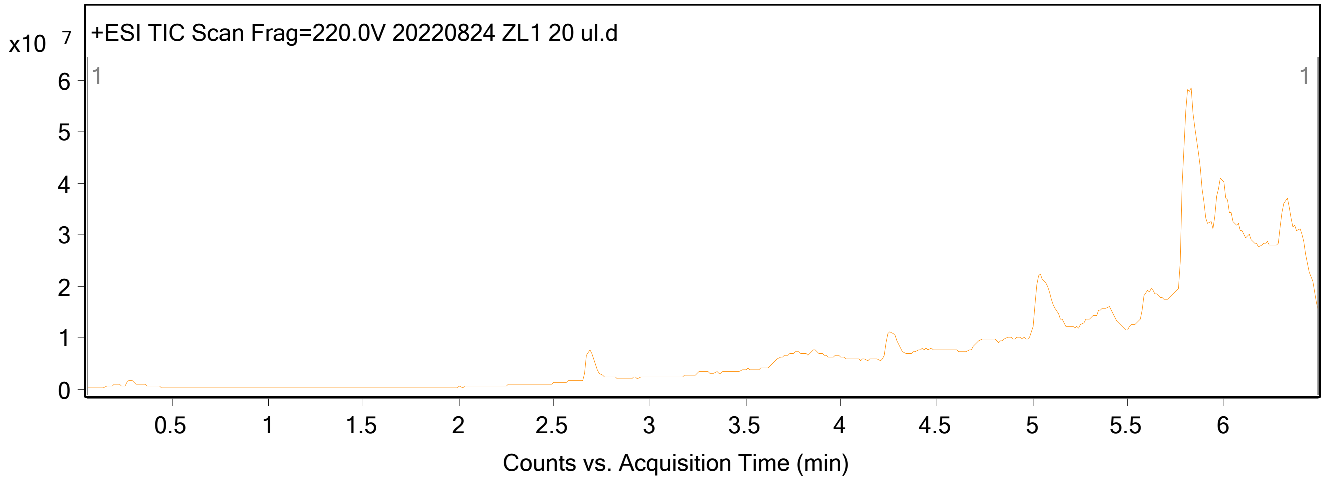

Qualitative Analysis Report

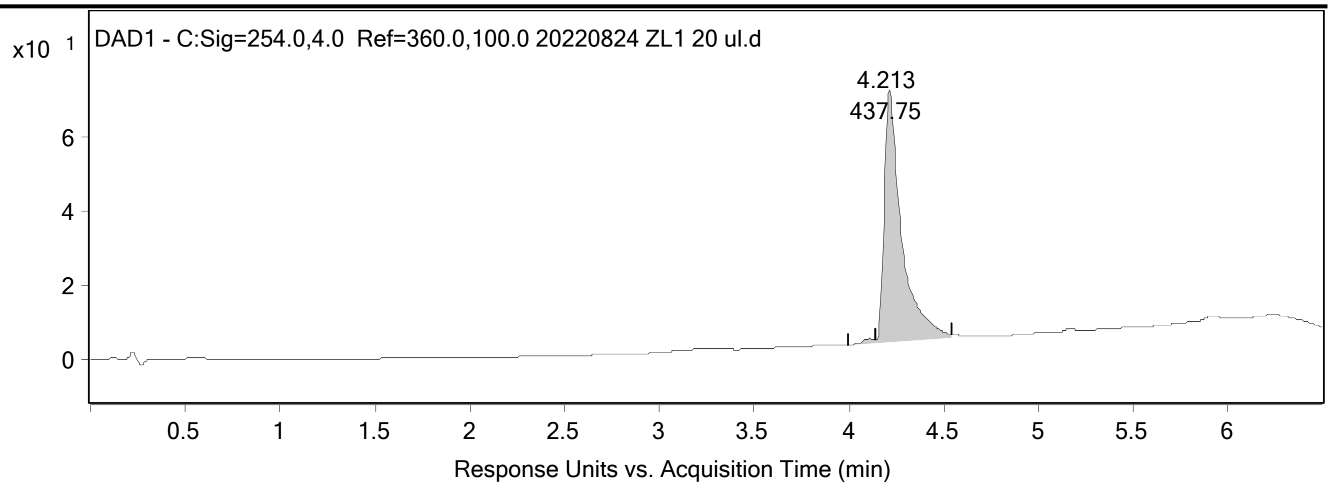

Integration Peak List

Peak	Start	RT	End	Height	Area	Area %
1	3.993	4.107	4.14	1.5	6.4	1.46
2	4.14	4.213	4.54	67.7	437.75	100

Spectra

Fragmentor Voltage	Collision Energy	Ionization Mode
220	0	ESI

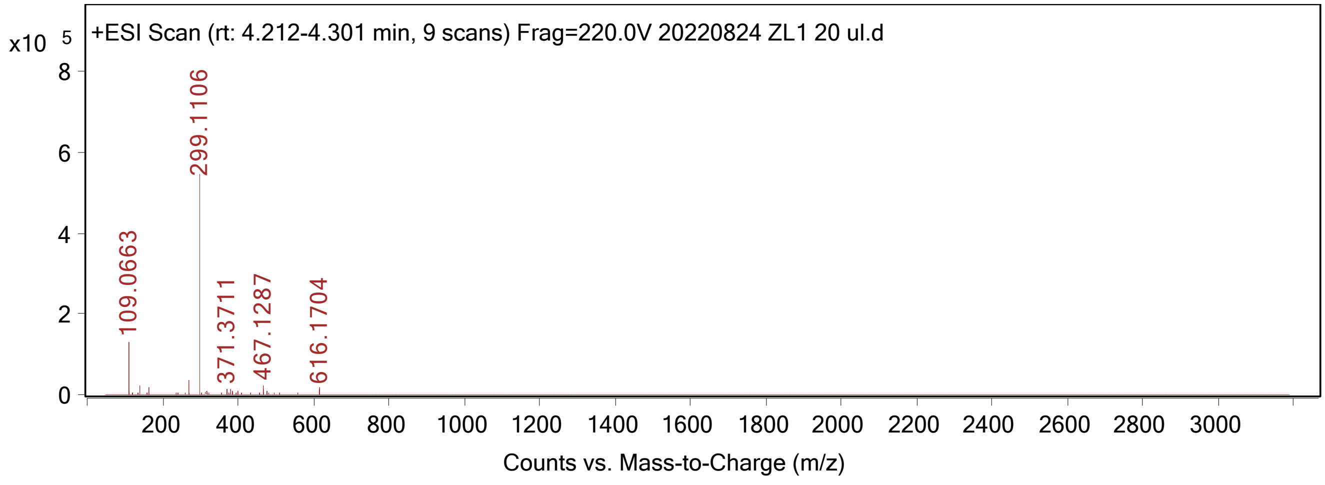

Peak List

m/z	z	Abund
109.0663	1	132829.47
137.0641		23591.36
163.0658		20465.17
268.2999		36770.89
299.1106	1	557872.44
300.1139	1	55106.45
371.3711		14502.39
382.4855	2	13468.01
467.1287	2	24927.58

616.1704	2	16712.19

--- End Of Report ---

ZL2
PROTON DMSO {C:\Bruker\TopSpin3.2} core_user 2

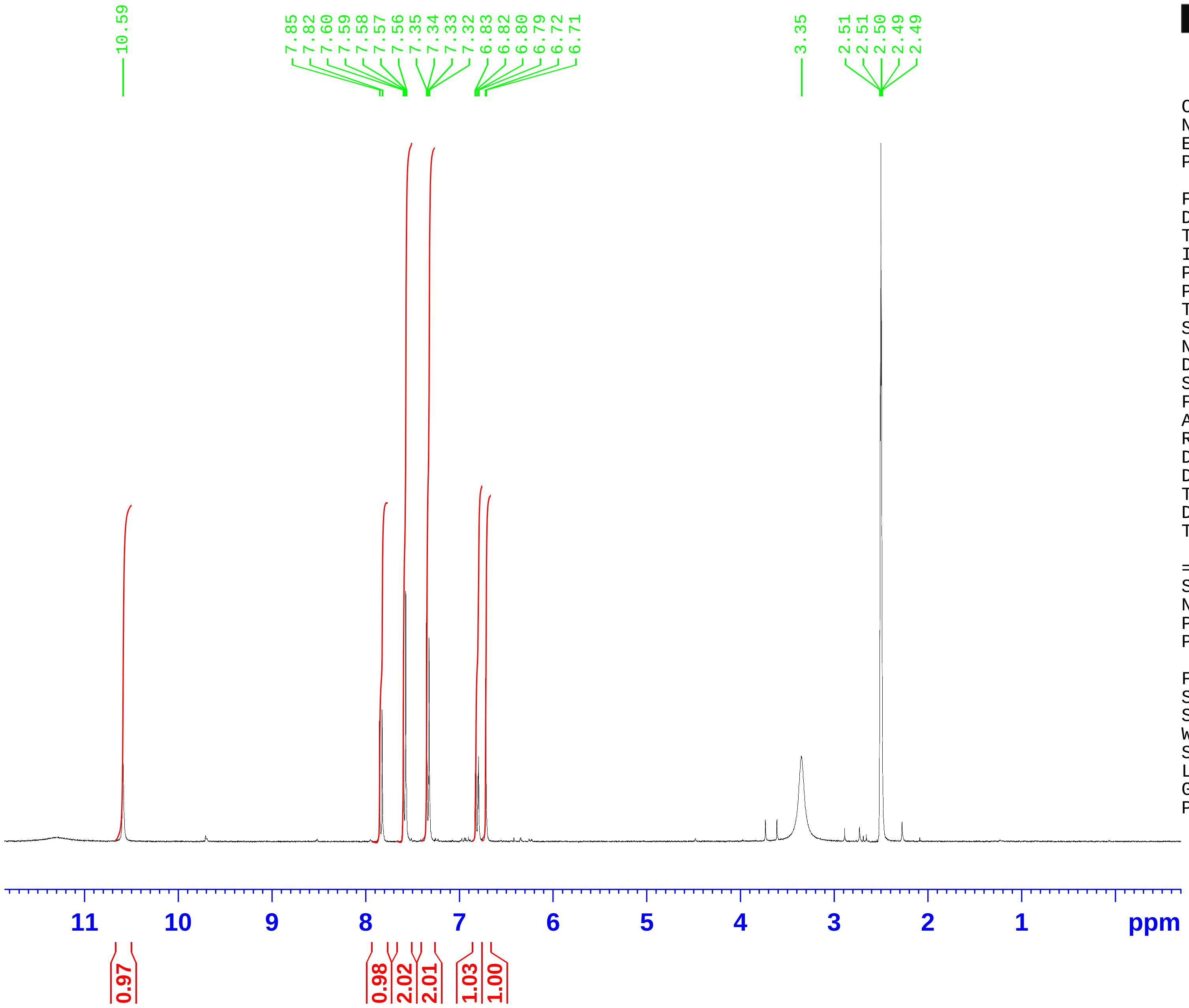

Current Data Parameters
NAME Aug19-2022
EXPNO 10
PROCNO 1

F2 - Acquisition Parameters
Date_ 20220819
Time 15.39
INSTRUM FOURIER300
PROBHD 5 mm DUL 13C-1
PULPROG zg30
TD 65536
SOLVENT DMSO
NS 16
DS 2
SWH 6103.516 Hz
FIDRES 0.093132 Hz
AQ 5.3687091 sec
RG 162.587
DW 81.920 usec
DE 6.50 usec
TE 294.3 K
D1 1.00000000 sec
TD0 1

======== CHANNEL f1 ========
SFO1 300.1818537 MHz
NUC1 1H
P1 14.20 usec
PLW1 9.00000000 W

F2 - Processing parameters
SI 65536
SF 300.1800001 MHz
WDW EM
SSB 0
LB 0.30 Hz
GB 0
PC 1.00

Qualitative Analysis Report

**Data File**	20220818 ZL2.d	**Sample Name**	
**Sample Type**	Sample	**Position**	P2-C1
**Instrument Name**	LC QTOF	**User Name**	
**Acq Method**	Small Molecule Training_0.6mLPerMin_7Min_30P of B.m	**Acquired Time**	8/18/2022 4:47:51 PM (UTC-04:00)
**IRM Calibration Status**	Success	**DA Method**	default.m
**Comment**			
**Sample Group**		**Info.**	
**Stream Name**	LC 1	**Acquisition Time (Local)**	8/18/2022 4:47:51 PM (UTC-04:00)
**Acquisition SW Version**	6200 series TOF/6500 series Q-TOF B.08.00 (B8058.0)	**QTOF Driver Version**	8.00.00
**QTOF Firmware Version**	20.698	**Tune Mass Range Max.**	3200

Chromatograms

Fragmentor Voltage 220 **Collision Energy** 0 **Ionization Mode** ESI

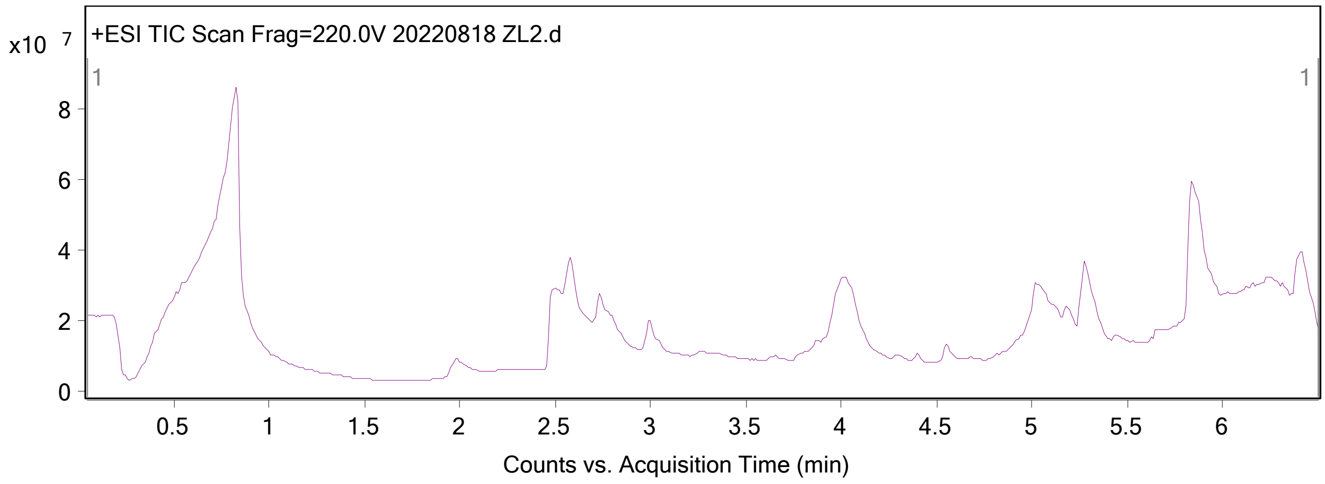

Qualitative Analysis Report

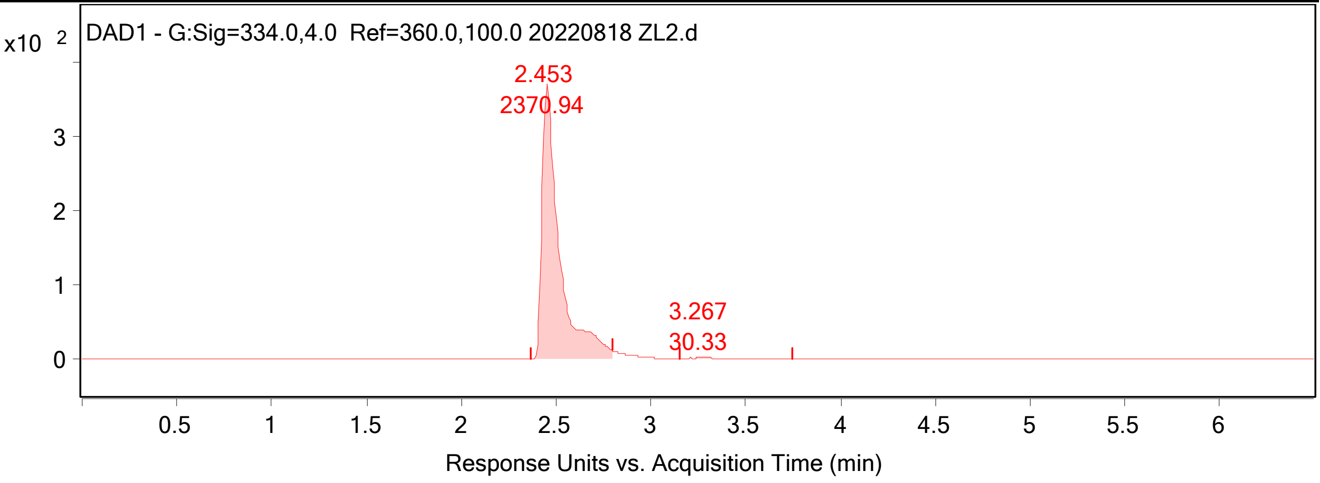

Integration Peak List

Peak	Start	RT	End	Height	Area	Area %
1	2.367	2.453	2.793	369.17	2370.94	100
2	3.153	3.267	3.747	2.63	30.33	1.28

Spectra

Fragmentor Voltage	Collision Energy	Ionization Mode
220	0	ESI

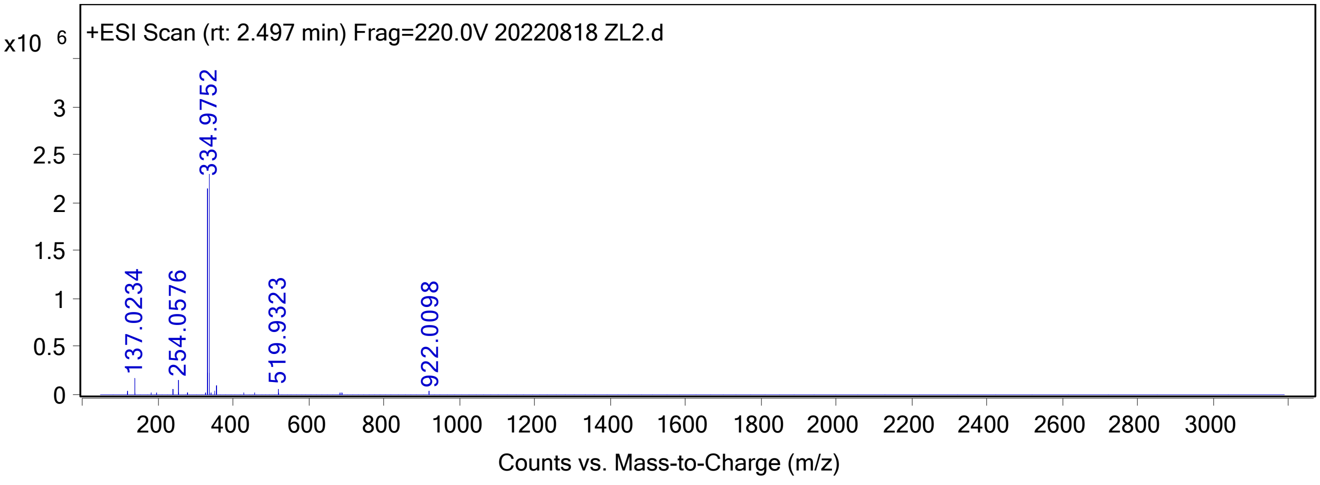

Peak List

m/z	z	Abund
137.0234		182898.71
254.0576		147626
332.9769	1	2210423.32
333.9798	1	230598.75
334.9752	1	2296122.76
335.978	1	235560.86
354.9578		100308.95
356.956		104969.98
518.9334	2	57522.26

519.9323	2	61013.51

--- End Of Report ---

ZL3
PROTON DMSO {C:\Bruker\TopSpin3.2} core_user 4

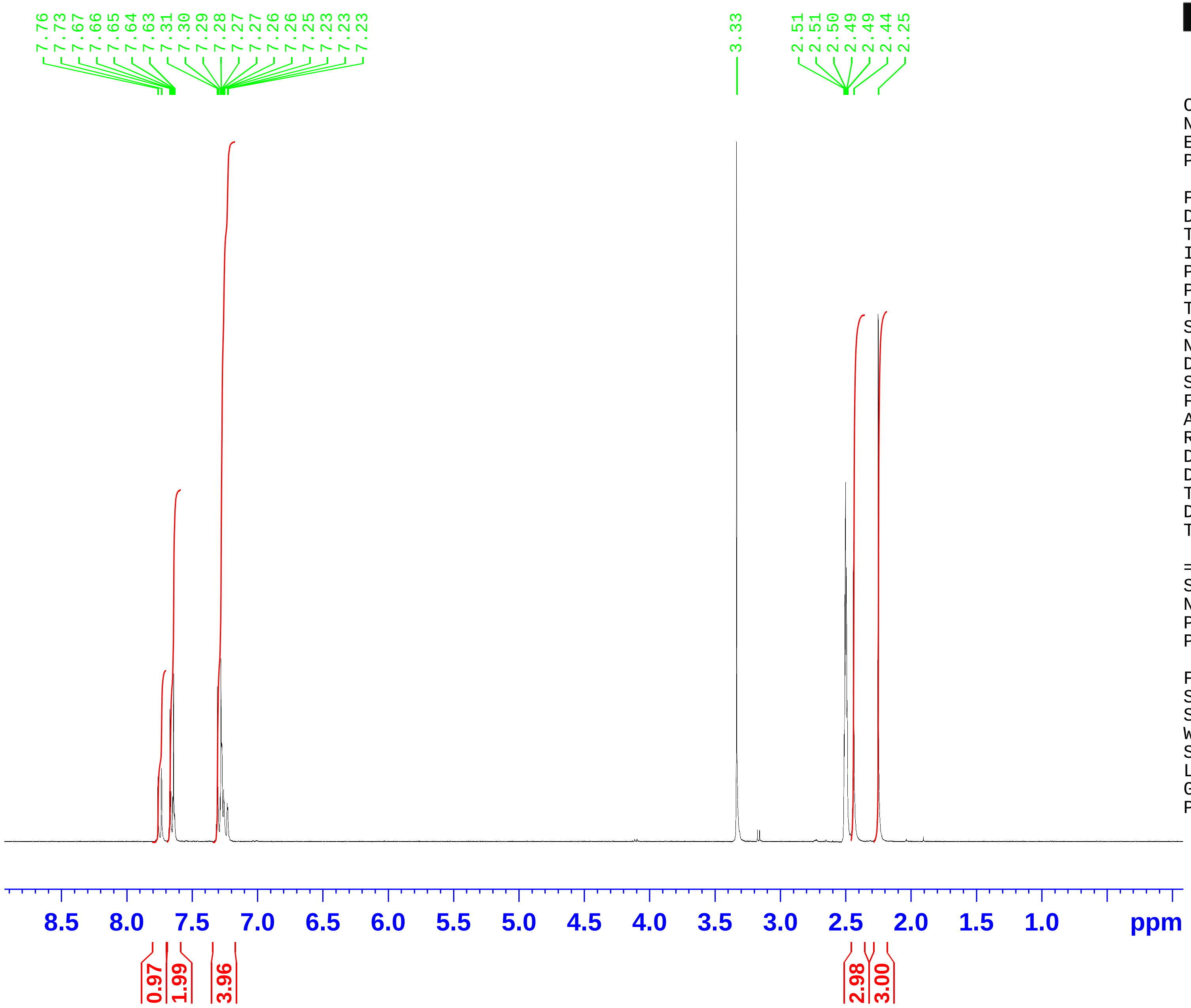

Current Data Parameters
NAME Aug19-2022
EXPNO 20
PROCNO 1

F2 - Acquisition Parameters
Date_ 20220819
Time 15.45
INSTRUM FOURIER300
PROBHD 5 mm DUL 13C-1
PULPROG zg30
TD 65536
SOLVENT DMSO
NS 16
DS 2
SWH 6103.516 Hz
FIDRES 0.093132 Hz
AQ 5.3687091 sec
RG 129.312
DW 81.920 usec
DE 6.50 usec
TE 294.4 K
D1 1.00000000 sec
TD0 1

======== CHANNEL f1 ========
SFO1 300.1818537 MHz
NUC1 1H
P1 14.20 usec
PLW1 9.00000000 W

F2 - Processing parameters
SI 65536
SF 300.1800002 MHz
WDW EM
SSB 0
LB 0.30 Hz
GB 0
PC 1.00

Qualitative Analysis Report

**Data File**	20220818 ZL3.d	**Sample Name**	
**Sample Type**	Sample	**Position**	P2-C2
**Instrument Name**	LC QTOF	**User Name**	
**Acq Method**	Small Molecule Training_0.6mLPerMin_7Min_30P of B.m	**Acquired Time**	8/18/2022 4:55:19 PM (UTC-04:00)
**IRM Calibration Status**	Success	**DA Method**	default.m
**Comment**			
**Sample Group**		**Info.**	
**Stream Name**	LC 1	**Acquisition Time (Local)**	8/18/2022 4:55:19 PM (UTC-04:00)
**Acquisition SW Version**	6200 series TOF/6500 series Q-TOF B.08.00 (B8058.0)	**QTOF Driver Version**	8.00.00
**QTOF Firmware Version**	20.698	**Tune Mass Range Max.**	3200

Chromatograms

Fragmentor Voltage 220 **Collision Energy** 0 **Ionization Mode** ESI

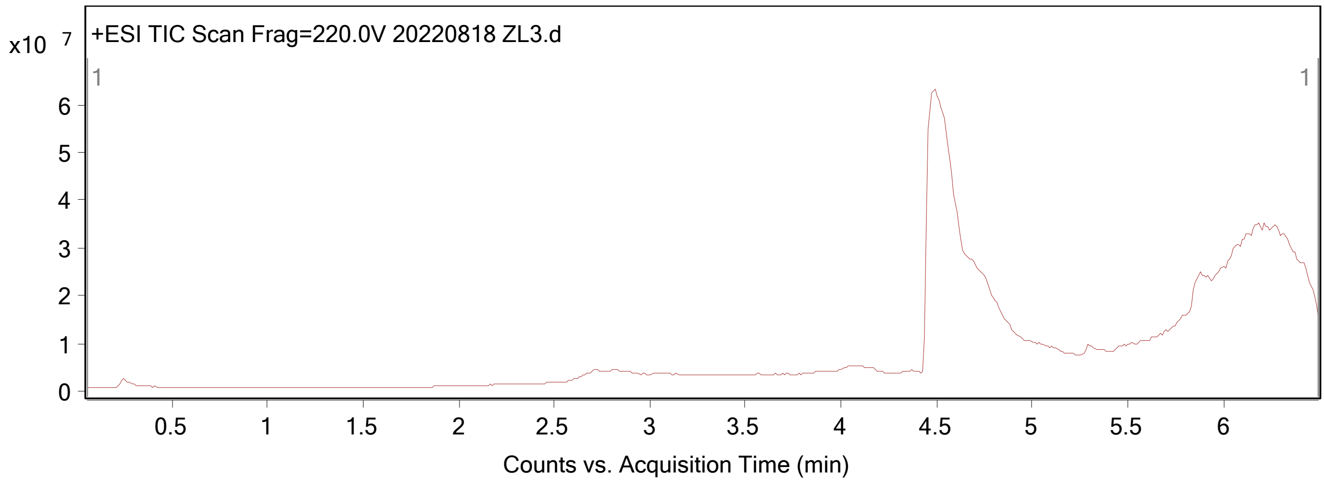

Qualitative Analysis Report

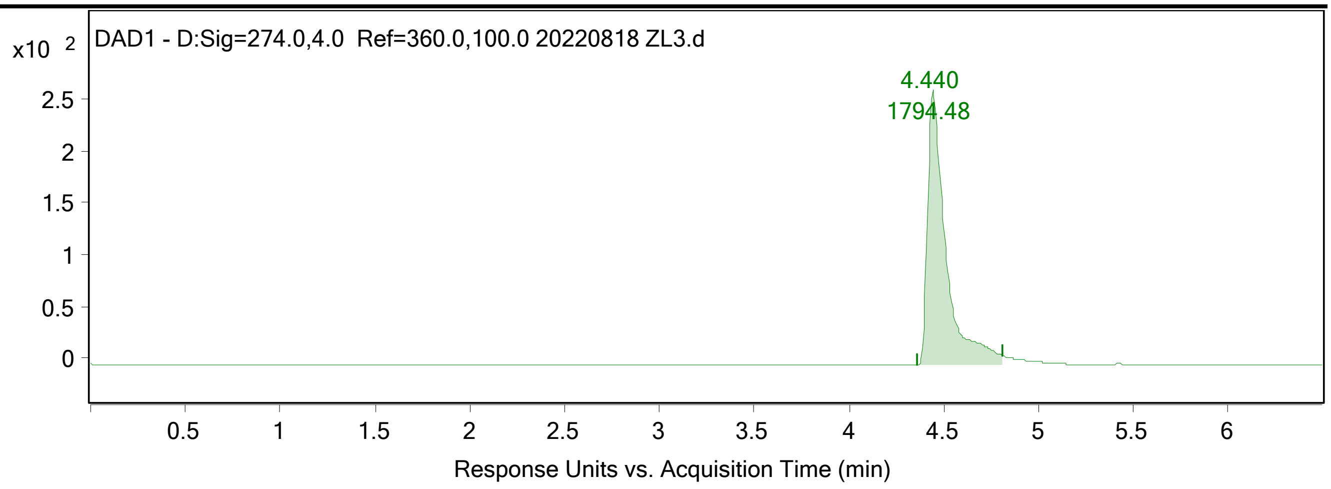

Integration Peak List

Peak	Start	RT	End	Height	Area	Area %
1	4.36	4.44	4.807	265.07	1794.48	100

Spectra

Fragmentor Voltage	Collision Energy	Ionization Mode
220	0	ESI

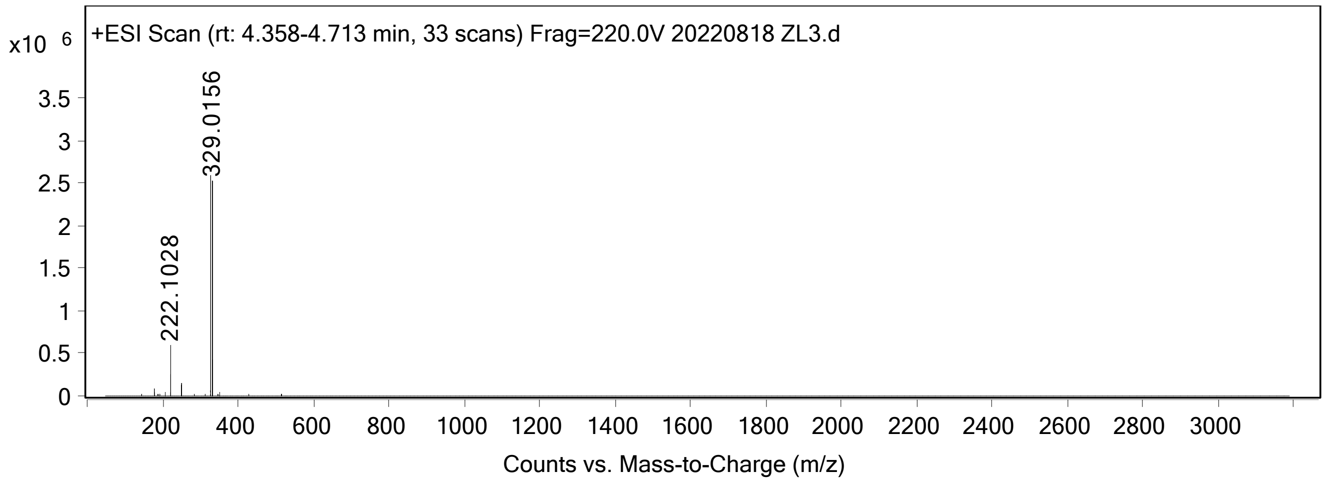

Peak List

m/z	z	Abund
178.0771		98261.64
221.0956		254636.19
222.1028	1	618397.51
223.106	1	52393.63
249.0898		170119.28
250.0969		124260.88
329.0156	1	2601066.55
330.0179	1	436239.48
331.0137	1	2603752.85
332.016	1	404439.9

--- End Of Report ---

ZL4.fid 1 1 "C:\Users\Lifan Zeng\Box Sync\Chemical Genomics\4, CGCF Projects\Medicinal Chemistry\202

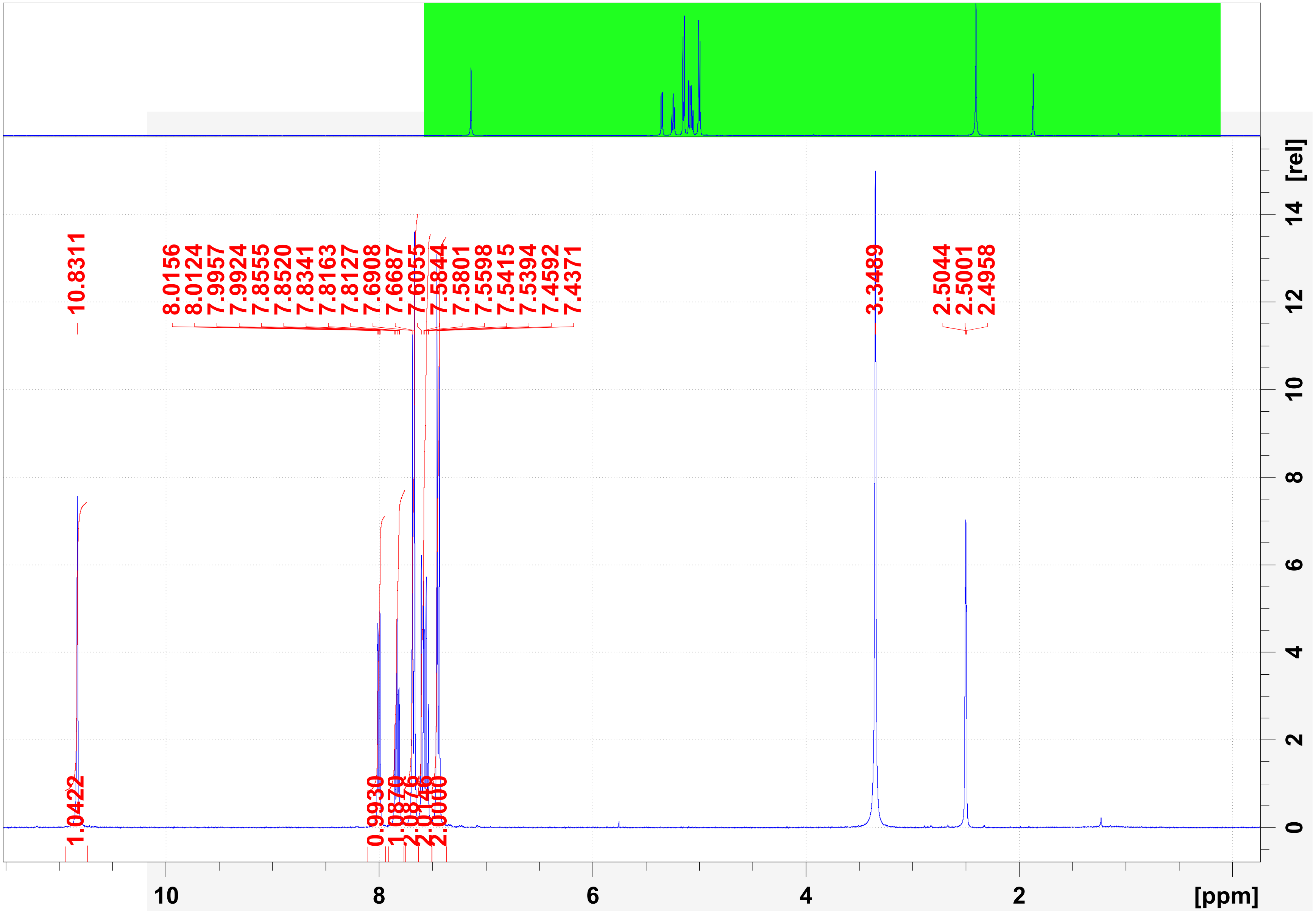

Qualitative Analysis Report

**Data File**	20221215 ZL4 110v.d	**Sample Name**	
**Sample Type**	Sample	**Position**	P2-B6
**Instrument Name**	LC QTOF	**User Name**	
**Acq Method**	Small Molecule Training_0.6mLPerMin_7Min 110V.m	**Acquired Time**	12/15/2022 4:10:17 PM (UTC-05:00)
**IRM Calibration Status**	Success	**DA Method**	default_small_molecule.m
**Comment**			
**Sample Group**		**Info.**	
**Stream Name**	LC 1	**Acquisition Time (Local)**	12/15/2022 4:10:17 PM (UTC-04:00)
**Acquisition SW Version**	6200 series TOF/6500 series Q-TOF B.08.00 (B8058.0)	**QTOF Driver Version**	8.00.00
**QTOF Firmware Version**	20.698	**Tune Mass Range Max.**	3200

Chromatograms

Fragmentor Voltage 110 **Collision Energy** 0 **Ionization Mode** ESI

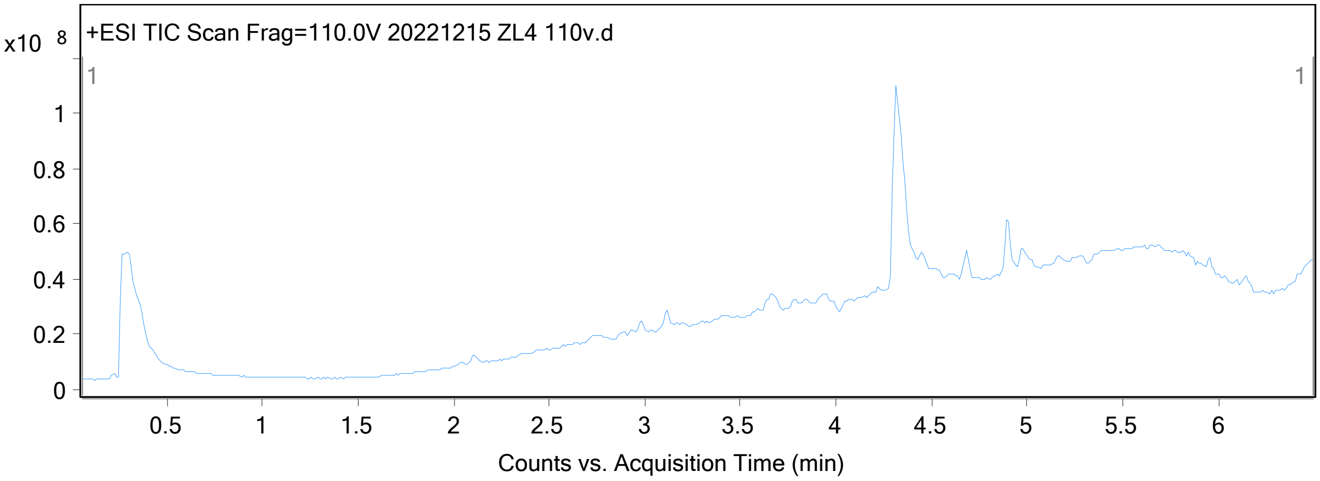

Qualitative Analysis Report

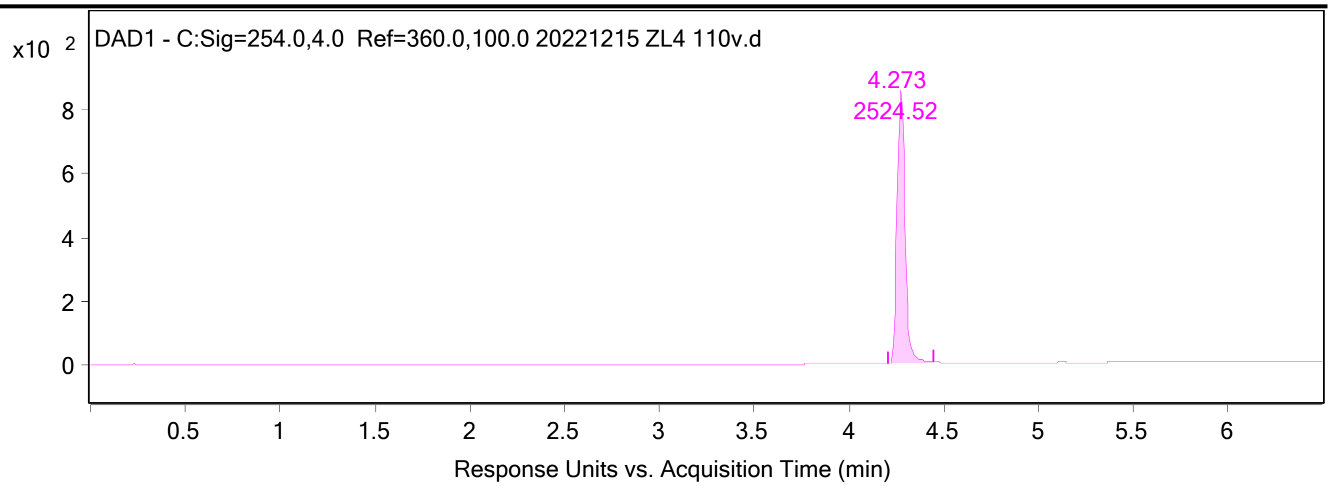

Integration Peak List

Peak	Start	RT	End	Height	Area	Area %
1	4.207	4.273	4.44	855.16	2524.52	100

Spectra

Fragmentor Voltage	Collision Energy	Ionization Mode
110	0	ESI

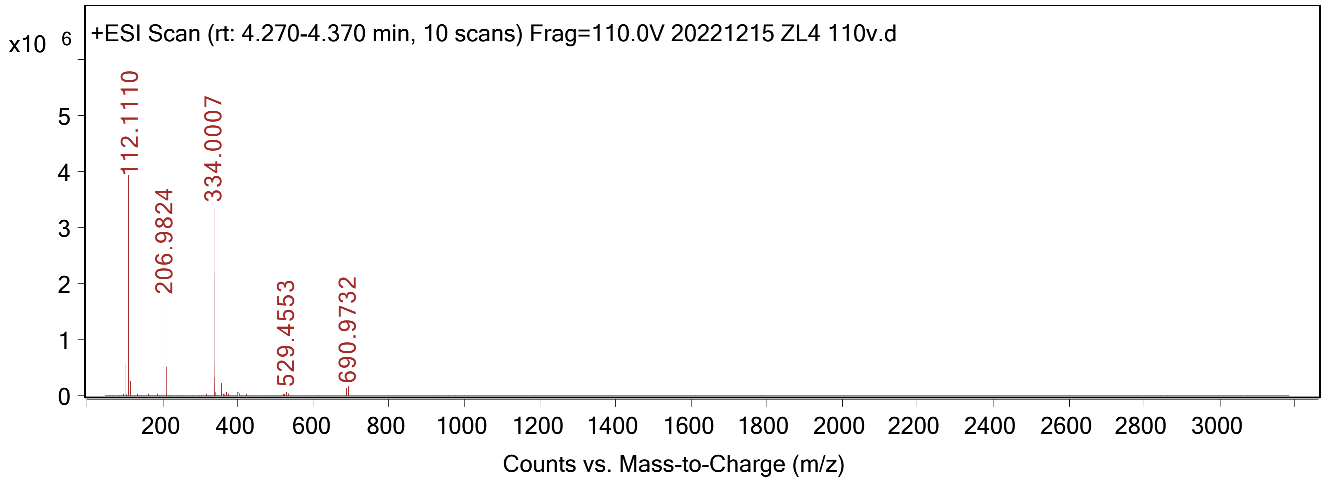

Peak List

m/z	z	Abund
102.1265		594376.6
112.111	1	4062442.06
113.1142	1	276376.21
206.9824	1	1733305.16
208.9796	1	528066.91
334.0007	1	3442261.73
335.0036	1	569281.04
335.9976	1	2249844.14
337.0009	1	324610.14
337.9957	1	324381.43

--- End Of Report ---
